# A Case of Competitive Aromatization vs. Sigmatropic [1,5]-Hydrogen Atom Migration in a 1,2,4-Cyclohexatriene Intermediate Derived from a Bis-Enyne Cyclization

**DOI:** 10.3390/molecules30122610

**Published:** 2025-06-16

**Authors:** Rong Tang, Qian Xu, Thomas R. Hoye

**Affiliations:** Department of Chemistry, University of Minnesota, Minneapolis, MN 55455, USA; tang0658@umn.edu (R.T.); xu000243@umn.edu (Q.X.)

**Keywords:** thermal rearrangements, strain-promoted reactions, tetradehydro-Diels–Alder (TDDA)

## Abstract

1,2,4-Cyclohexatrienes are strained, reactive intermediates often formed by the tetradehydro-Diels–Alder (TDDA) reaction of a conjugated enyne bearing a tethered alkyne as the enynophile. The ene component is commonly the π-bond of an aromatic group. In this focused study, we investigated the reactivity of a symmetrical substrate in which the pair of terminal ene moieties were simple 2-propenyl groups. The intermediate 1,2,4-cyclohexatriene, now bearing a 5-isopropenyl group, underwent competitive aromatization (the most usual outcome of the strain-relieving event of the cyclohexatriene), along with an intramolecular [1,5]-hydrogen atom migration, ultimately producing a non-benzenoid, pyrrole derivative. This represents a previously unknown process for a 1,2,4-cyclohexatriene derivative. Mechanistic aspects of the competitive processes were revealed by experiments performed in the presence of various protic additives (MeOD and BHT).

## 1. Introduction

**Figure 1:**
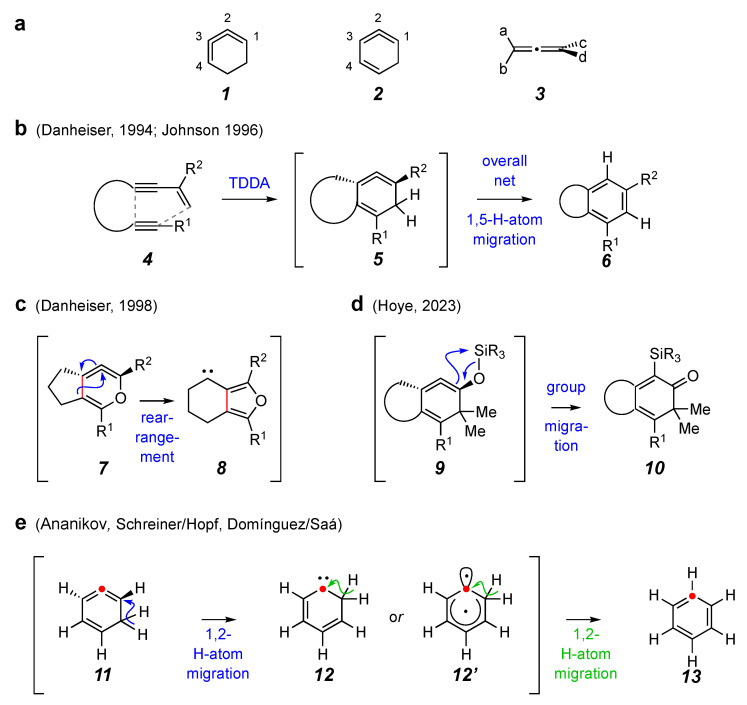
(**a**) Strained, cyclic cyclohexatrienes (**1** and **2**) vs. the strain-free parent, propa-1,2-diene (also known as allene, **3**), whose most stable geometry has orthogonal (90°) a-C-b and c-C-d planes. (**b**) Formation of 1,2,4-cyclohexatriene derivatives by a tetradehydro-Diels–Alder (TDDA) reaction and their aromatization to benzene derivatives by formal [1,5]-hydrogen atom migration [9,10]. (**c**) Relief of ring-strain by skeletal rearrangement [11]. (**d**) Relief of ring-strain by migration of an exocyclic group [12]. (**e**) Computed lowest energy pathway for the net [1,5]-hydrogen atom migration via two, sequential (blue then green) 1,2-H-atom migrations [13,14,15].

1,2,3-Cyclohexatriene (**1**, Figure 1a), a reactive intermediate and isomer of benzene, was first formed and intercepted in 1990 [1]. The isomeric 1,2,4-cyclohexatriene (**2**) was first generated and trapped in 1992 [2]. These species and their derivatives remain of contemporary interest [3,4]. Members of this class of compounds are highly reactive because they house a highly strained cyclic allene within a small ring. The strain energies in **1** and **2** have been evaluated computationally and are estimated to be 50 and 34 kcal mol^−1^, respectively [5]. The two pairs of terminal substituents on acyclic allenes (1,2-propadiene derivatives, **3**) ideally, of course, have an orthogonal relationship. The constraints of any rings (with the exception of very large ones) that house a cyclic allene dictate that the 1,2-propadiene unit is unable to achieve that ideal geometry, hence introducing increasing amounts of strain for lower homologs within smaller rings [5,6].

1,2,4-Cyclohexatriene derivatives are often formed as transient intermediates in, for example, net (4 + 2) cycloadditions between an alkyne and a 1,3-enyne in a process that can be categorized as a tetradehydro-Diels–Alder (TDDA) reaction [7,8] (cf. **4** to **5**, Figure 1b [9,10]). The transient 1,2,4-triene is known to undergo several types of processes to relieve the strain of the significantly twisted allene that it houses. Hydrogen atom migration that results in a benzenoid product (**5** to **6**, Figure 1b) is most common. Rarer are processes that result in non-aromatized products (**7** to **8**, [11] Figure 1c; or **9** to **10**, [12] Figure 1d).

## 2. Results and Discussion

Of the three modes of reaction shown in Figure 1b–d, the isomerization of the cyclic allene to its isomeric benzenoid product (cf. **5** to **6**) is by far the most common and, therefore, well studied. The generally accepted mechanism of the aromatization of 1,2,4-cyclohexatriene (and its derivatives, at least under conditions where there is no proton source in the reaction medium), is portrayed in Figure 1e [**11** to benzene (**13**)]. Computations [13,14,15] suggest that this thermal-only pathway involves two consecutive 1,2-H-atom shifts rather than a single [1,5]-H-atom migration. The first 1,2-shift gives a species (carbene or orthogonal diradical, cf. **12**/**12′**) that undergoes a rapid, second C–H insertion/migration event.

**Figure 2:**
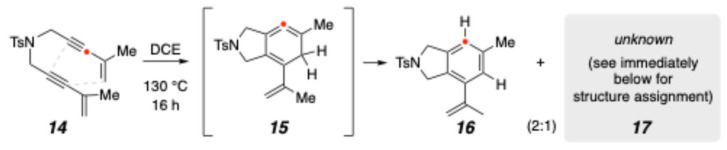
The unexpected formation of **17**, an isomer of the aromatized TDDA product, **16**.

In the course of studying the thermal cyclization chemistry of bis-enyne **14**, we observed, in addition to the normal aromatized benzenoid TDDA product, **16**, an unexpected side product [16], **17** (Figure 2). The ratio of **16**:**17** under these conditions was ca. 2:1. Although this ratio changed under other reaction conditions (see below), **17** was never the major product. The assignment of the structure of the expected **16** was straightforward from analysis of its ^1^H and ^13^C 1D and 2D NMR spectra (see Supporting Information (SI)). However, the structure of **17** was not at all clear. Only after determining its structure would we be in a position to hypothesize a pathway for its formation.

It was straightforward to confirm (GCMS) that **17** had the same mass as **16** (i.e., they were isomers). The chromatographic behaviors on silica gel of **16** and **17** were nearly the same, but by shaving the fractions eluting from an MPLC separation, we were able to obtain **17** in a 96:4 ratio with **16**. In the proton NMR spectrum of **17**, it was clear that the NTs moiety was intact. It also contained three singlets, whose chemical shifts (δ 7.04, 6.83, and 6.07 ppm) were indicative of protons attached to sp^2^ hybridized carbon atoms. Another significant difference in the spectrum (vs. that of **16**) was the presence of three, rather than two, methyl singlets (in addition to the Ts-Me), judged to be either allylic or benzylic in nature. The remaining protons were a singlet methylene pair (2.99 ppm) that, among other things, indicated that **17** possessed an achiral structure.

**Figure 3:**
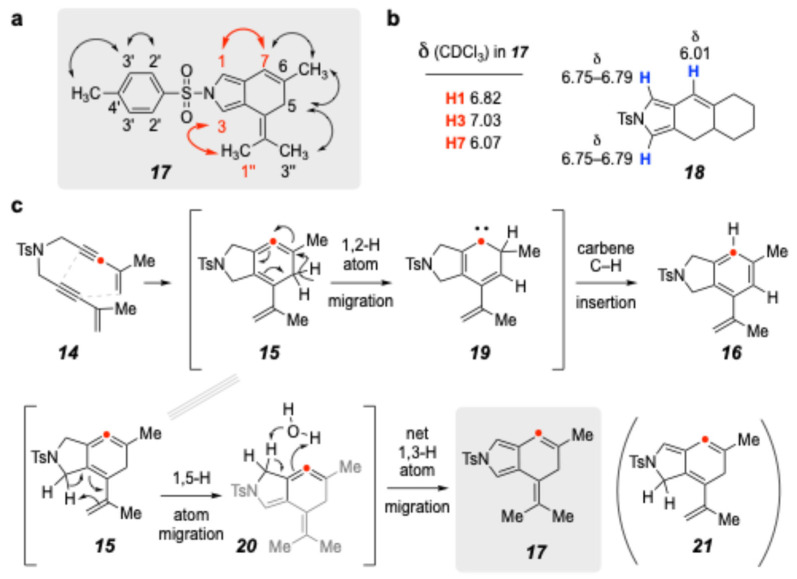
(**a**) Nuclear Overhauser enhancements in **17**. (**b**) Chemical shifts of C_sp2_ protons in **18** [17] vs. **17**. (**c**) Proposed mechanism: branching from cyclic allene **15** to account for formation of **16** and **17**.

The NOESY NMR spectrum was particularly informative; enhancements (double-headed arrows in Figure 3a) led us to propose structure **17** as the identity of the unknown. More specifically, the NOEs between what are assigned there as H1/H7 and H3/H1′′ (red) were quite diagnostic. To further support this assignment, we located in the literature compound **18** [17], a structurally analogous, fused *N*-tosyl pyrrole that possesses quite similar chemical shifts of the indicated protons in **18** vs. the analogous protons in **17** (Figure 3b). We also carried out a DP4+ analysis [18] of the NMR chemical shifts of the protons and carbons in **17** vs. an isomeric alkene having the endocyclic cyclohexene double bond between C5 and C6; this analysis also supported the assignment of **17** (see SI). Finally, we performed resolution enhancement of the (many) “singlets” in **17**, mentioned in the above paragraph; this revealed (many) small coupling constants that further reinforced the structure assignment (see *J* values in the ^1^H NMR line listings in Section 3).

With the structure of the unusual side product **17** established, we were obliged to propose a mechanism to account for its formation, especially so because, as far as we are aware, it represents an unprecedented type of transformation for a product arising from a TDDA reaction via a 1,2,4-cyclohexatriene-containing intermediate such as **5**. We show in Figure 3c a proposed pathway that branches following the initial formation of the intermediate **15** from an initial TDDA cycloisomerization. The 1,2-hydrogen atom migration to **19** and final carbene C–H insertion account for the major product, **16**. But in addition, the isopropenyl alkene in intermediate **15** is poised to undergo a sigmatropic rearrangement, namely [1,5]-hydrogen atom migration to produce **20**. A final net 1,3-hydrogen atom migration (tautomerization) of the strained cyclic allene in **20** would establish the pyrrole ring in the minor product, **17**. As indicated by the arrows in structure **20**, there is the possibility that this aromatization could be mediated (catalyzed) by a protic molecule (e.g., water) acting as a proton shuttle; this conversion could be either concerted or stepwise, with the latter following an initial protonation of the central carbon of the allene.

It should be noted that allenes are known to undergo reaction with Brønsted acids via kinetic protonation at either their terminal or central atoms to give vinylic or allylic carbocations, respectively [19]. Particularly relevant here are the differences seen between unstrained, acyclic allenes vs. strained, cyclic allenes. The former allenes tend to prefer kinetic protonation at a terminal carbon atom, although even for this class of unstrained allenes, this selectivity can often be reversed by the nature of substituents on the terminal atoms. In contrast, strained, cyclic allenes, tend to protonate preferentially at their central carbon atom, with there being a reduced amount of twist needed to attain the more stable allylic cation. An early report that brought this difference to light described the addition of HCl to cyclonona-1,2-diene to give only 3-chlorocyclononene [20]. The 1983 review by Smadja [19] contains a collection of many dozens of examples of “protonation of allenic derivatives” from which these general trends can be gleaned.

Several experiments were performed to probe the possible role that a hydroxyl-containing species might be playing. Saá and coworkers have used CD_3_OD (or CH_3_OD) as an additive and observed incorporation of a deuterium atom at the central carbon of TDDA-derived cyclic allene intermediates generated in their experiments (further discussed below) [15,21]. This established that a protic additive provided a pathway for aromatization that was faster than the unimolecular series of 1,2-H migrations computed for the parent system (cf. Figure 1e). We performed an analogous experiment with our substrate **14**. Heating the dienyne **14** in a mixture of DCE and CD_3_OD (90:10, v:v) at 130 °C for 16 h led to the formation of **16-d** and **17-d**, deuterated to ca. 91% and 90% (^1^H NMR) and in a ratio of 2:1, that ratio being the same as the reaction performed without added methanol. An analogous experiment using CH_3_OD as the additive also showed that both **16-d** and **17-d** were produced, indicating that the hydroxyl group, and not the methyl group, was the source of the hydrogen incorporated into each of those products.

**Figure 4:**
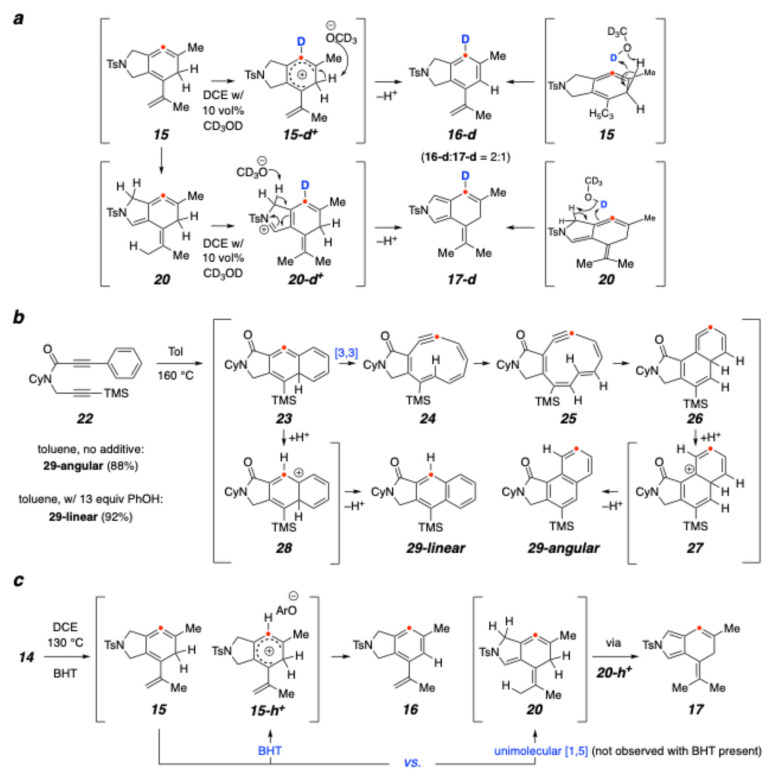
(**a**) Possible mechanisms for the key steps to produce the deuterated products **16-d** and **17-d**. (**b**) Relevant results from Saá and coworkers showing the effect of phenol product distribution from the TDDA reaction of **22**. (**c**) The more acidic protic additive BHT quickly tautomerizes the 1,2,4-cyclohexatriene **15** to **16** before **15** has time to isomerize to **20** via [1,5]-hydrogen atom migration.

These labeling experiments do not differentiate between the possible stepwise (via **15-d^+^** or **20-d^+^**) vs. concerted net 1,3-tautomerizations shown in Figure 4a. A fuller discussion of the Saá experiments is instructive because they shed light on this question. As outlined in Figure 4b, heating the TDDA substrate **22** in toluene alone produced solely the naphthalene product **29-angular**. This can be rationalized by the sequence of electrocyclic and isomerization events commencing from the initial cyclic allene **23** and proceeding through **24**–**26**. Computations supported the main features of this pathway, although whether the final aromatization occurred via sequential intramolecular 1,2-H shifts (cf. Figure 1e) or a protic molecule-assisted event (e.g., via **27**) was not discerned. However, the role of the protic additive phenol was clearly demonstrated because, when present, it resulted in the formation of **29-linear** as the only product. This is explained by protonation of allene **23** by PhOH more quickly than the rate of unimolecular ring-opening of **23** to **24**.

We performed a similar experiment with **14** using butylated hydroxytoluene (BHT) as a phenolic, protic additive. In the presence of 0.1 equivalent of BHT, the ratio of **16** to **17** changed to 6:1. Using 1.0 equivalent of BHT, we observed formation of only the aromatic product **16**. This suggests that even with the use of a small amount of BHT, the cyclic allene was protonated to give **15-h^+^** at a rate competitive with its [1,5]-H atom migration, and that the larger amount of BHT promoter fully suppressed the [1,5]-H atom migration. Recall that even in the presence of 10 vol% of methanol, the ratio of products **16** to **17** was the same as when no methanol was present. The different effect of the BHT additive is most likely a reflection of its greater acidity relative to that of methanol (MeOH pKa_(DMSO)_ = 29.0 [22], PhOH pKa_(DMSO)_ = 18.0 [23]), thereby accelerating the production of **15-h^+^**.

**Figure 5:**
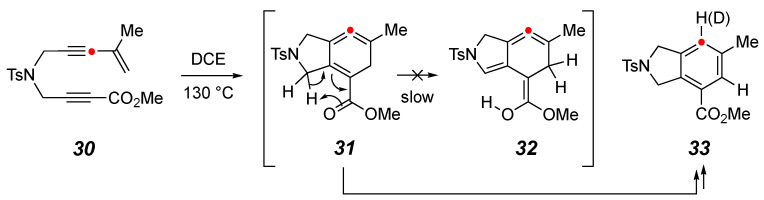
Substrate **30** containing only one enyne moiety leads to **33** as the only isolable product, suggesting that the [1,5]-hydrogen atom migration in **31** (to give, transiently, **32**) is not occurring to a significant extent.

Finally, we prepared the ynoate/enyne substrate **30** (Figure 5). When heated, it reacted at a similar rate to that of **14**. It produced the aromatized compound **33** as the major product. As expected, when this reaction was performed in the presence of CD_3_OD, a deuterium atom was incorporated into the product **33-d**. In principle, the carbonyl group of the ester in the intermediate cyclic allene **31** could have engaged in a [1,5]-hydrogen atom migration to give **32**. However, the relative instability of the enol tautomer of an ester makes this a sufficiently high-barrier process, so there is little opportunity for that to occur enroute to a pyrrole derivative analogous to **17**. This further emphasizes the unique role that the isopropenyl substituent is playing in diverting the reactivity of **15**.

## 3. Experimental Section

### 3.1. Chemistry

#### 3.1.1. General Experimental Protocols

^13^C and ^1^H NMR spectra were obtained using a Bruker HD-500 spectrometer. All spectra were recorded in CDCl_3_. The reported proton chemical shifts are referenced to CHCl_3_ in (δ = 7.26 ppm) in CDCl_3_. Resonances in the line listings of ^1^H NMR spectral data are given in this format: chemical shift (δ, ppm) [multiplicity, coupling constant(s) *J* (in Hz), integral value (to the nearest integer)]. First-order coupling constants were extracted using methods we have described elsewhere [24,25]. An nfom, nfod, or nfot refers to a non-first order multiplet, doublet, or triplet in a ^1^H NMR spectrum. Coupling constants for these resonances are given as apparent values (*J_app_*); for example, the observed spacing between the two most intense lines in an nfod is the value of *J_o_* + *J_p_* and not the true doublet coupling constant. The reported carbon chemical shifts in ^13^C spectra are referenced to CDCl_3_ (δ = 77.16 ppm). Infrared spectra were recorded using a using a Bruker Alpha II Spectrometer. The data were collected in attenuated total reflectance (ATR) mode. Samples were measured as thin films that were deposited onto a diamond ATR window by evaporative loss of solvent, often CDCl_3_. Absorption maxima are given as cm^−1^. High-resolution mass spectrometry (HRMS) data were collected in the ESI mode with a Thermo Orbitrap Velos instrument having mass accuracy of ≤3 ppm. Pierce^TM^ LTQ was used as an external calibrant. Samples were injected directly into the ion source. Melting points were measured on a Köfler hot-stage and polarizing microscope and are uncorrected. New substances were often purified by medium-pressure liquid chromatography (MPLC) at 25–200 psi. Silica gel columns (hand-packed, normal-phase, Teledyne RediSep Rf Gold^®^, 20–40 μm, 60 Å pore size) were used. The unit was assembled from a Waters HPLC pump (model 510) affixed to a Waters differential refractive index detector (R401). Preparative flash column chromatography was performed on columns hand-packed with Agela silica gel (230–400 mesh). Thin-layer chromatography (TLC) was carried out on silica gel-coated plates (plastic-backed), visualized by UV light and/or by staining with a solution of potassium permanganate. Reaction temperatures refer to the temperature of a pre-equilibrated and external heating oil bath. Any reaction performed at a temperature higher than that of the reaction solvent’s boiling point was carried out in a threaded culture tube, which was closed with an inert Teflon^®^-lined screw-cap.

#### 3.1.2. Synthesis of Precursors 14 and 30 (Structures of Intermediates Not Specifically Shown in Any of Figure 1, Figure 2, Figure 3, Figure 4 and Figure 5 Are Given S#s Here)

*N,N*-bis(4-hydroxy-4-methylpent-2-yn-1-yl)-4-methylbenzenesulfonamide (**S2**). In a 100 mL round-bottomed flask equipped with a magnetic stir bar, 4-methyl-*N*,*N*-di(prop-2-yn-1-yl)benzenesulfonamide (**S1** [26], 2.3 g, 9.4 mmol, 1 equiv) was added. THF (47 mL) was added at −78 °C under an atmosphere of N_2_. *n*-BuLi (7.9 mL, 2.5 M in hexanes, 2.1 equiv) was added dropwise. After 1 h of stirring at −78 °C, acetone (2.1 mL, 28 mmol, 3 equiv) was added dropwise. The reaction mixture was stirred at −78 °C for another 1 h. The reaction was quenched with saturated NH_4_Cl aqueous solution. THF was removed under vacuum, and the aqueous layer was extracted with DCM, washed with brine, dried (Na_2_SO4), concentrated, and purified by flash column chromatography (10:1 to 1:1 Hex:EtOAc) to give **S2** (2.9 g, 8.0 mmol, 85%) as a white solid. mp: 98–100 °C. ^1^H-NMR (500 MHz, CDCl_3_): δ 7.76 (nfod, *J_app_* = 8.3 Hz, 2H), 7.33 (nfod, *J_app_* = 8.0 Hz, 2H), 4.17 (s, 4H), 2.43 (s, 3H), 1.89 (s, 2H), and 1.37 (s, 12H). ^13^C-NMR{^1^H} (126 MHz, CDCl_3_): δ 144.0, 136.1, 129.7, 128.3, 90.5, 74.9, 65.1, 37.1, 31.2, and 21.6. IR (neat): ν_max_ 3524 (br O-H), 3265 (br O-H), 2981 (CH_3_), and 2924 (CH_2_) cm^−1^. HRMS (ESI-TOF): Calcd for C_19_H_26_NO_4_S^+^ [M + H]^+^, 364.1577; found, 364.1560 (9%). Calcd. for 346.1471 C_19_H_24_NO_3_S^+^ [M + H^+^ – H_2_O]; found, 346.1455 (100%).

4-Methyl-*N,N*-bis(4-methylpent-4-en-2-yn-1-yl)benzenesulfonamide (**14**). In a 100 mL round-bottomed flask, equipped with a magnetic stir bar, **S2** (790 mg, 2.2 mmol, 1 equiv) was added. DCM (13 mL) was added under a N_2_ atmosphere. To this solution, triethylamine (10.6 mL, 76 mmol, 35 equiv) was added at 0 °C. Methanesulfonic anhydride (6.7 g, 38 mmol, 18 equiv) dissolved in DCM (26 mL) was added dropwise. The reaction mixture was stirred at RT for 2.5 h. The reaction mixture was quenched with water and extracted with DCM. The combined organic layers were washed with brine, dried (Na_2_SO_4_), filtered, and concentrated; and the residue was purified by flash column chromatography (10:1 Hex:EtOAc) to give **14** (511 mg, 1.6 mmol, 72%) as a pale-yellow oil. ^1^H-NMR (500 MHz, CDCl_3_): δ 7.76 (nfod, *J_app_* = 8.0 Hz, 2H), 7.31 (nfod, *J_app_* = 8.2 Hz, 2H), 5.19 (br s, 2H), 5.13 (br s, 2H), 4.29 (s, 4H), 2.42 (s, 3H), and 1.76 (br s, *J* = 1.3 Hz, 6H). ^13^C-NMR{^1^H} (126 MHz, CDCl_3_): δ 143.8, 135.6, 129.7, 128.1, 126.1, 122.5, 87.0, 80.8, 37.3, 23.2, and 21.6. IR (neat): ν_max_ 3003 (C_sp2_-H), 2925 (H_2_C_sp3_-H), and 1598 (C=C) cm^−1^. HRMS (ESI-TOF): Calcd for C_19_H_22_NO_2_S^+^ [M + H]^+^ 328.1366; found 328.1353 (100%).

4-Methyl-*N*-(4-methylpent-4-en-2-yn-1-yl)-*N*-(prop-2-yn-1-yl)benzenesulfonamide (**S4**). In a culture tube, equipped with a magnetic stir bar, *N*-(4-hydroxy-4-methylpent-2-yn-1-yl)-4-methyl-*N*-(prop-2-yn-1-yl)benzenesulfonamide (**S3** [27], 305 mg, 1 mmol, 1 equiv) was added. DCM (5.9 mL) was added under an atmosphere of N_2_. Triethylamine (2.4 mL, 17.6 mmol, 17.6 equiv) was added at 0 °C. Methanesulfonic anhydride (1.5 g, 8.8 mmol, 8.8 equiv) was added dropwise. The reaction mixture was stirred at RT for 1 h and then poured into ice-cold concentrated aqueous HCl (2 mL). The aqueous layer was extracted with DCM. The combined organic layers were washed with brine, dried (Na_2_SO_4_), filtered, concentrated, and purified by MPLC (15:1 Hex:EtOAc) to give **S4** (162.88 mg, 0.56 mmol, 57%) as a pale-yellow oil. ^1^H-NMR (500 MHz, CDCl_3_): δ 7.72 (nfod, *J_app_* = 8.4 Hz, 2H), 7.29 (nfod, *J_app_* = 8.1 Hz, 2H), 5.15 (dq, *J* = 1.6, 1.6 Hz, 1H), 5.07 (dq, *J* = 1.8, 1.0 Hz, 1H), 4.29 (s, 2H), 4.13 (dd, *J* = 2.5, 0.7 Hz, 2H), 2.41 (t, *J* = 0.9 Hz, 3H), 2.16 (t, *J* = 2.5 Hz, 1H), and 1.71 (dd, *J* = 1.6, 1.1 Hz, 3H). ^13^C{^1^H} (126 MHz, CDCl_3_): δ 144.0, 135.4, 129.7, 128.1, 126.0, 122.5, 87.2, 80.4, 76.6, 74.0, 37.1, 36.5, 23.2, and 21.7. IR (neat): ν_max_ 3278 (C_sp_-H), 2982 (H_2_C_sp3_-H), 2927 (RHC_sp3_-H), and 1597 (C=C) cm^−1^. HRMS (ESI-TOF): Calcd for C_16_H_18_NO_2_S^+^ [M + H]^+^ 288.1053; found 288.1060 (100%).

Methyl 4-{[4-Methyl-*N*-(4-methylpent-4-en-2-yn-1-yl)phenyl]sulfonamido}but-2-ynoate (**30**). In a 10 mL round-bottomed flask, equipped with a magnetic stir bar, **S4** (100 mg, 0.35 mmol, 1 equiv) was added. THF (3.5 mL) was added under a N_2_ atmosphere. *n*BuLi (0.17 mL, 0.42 mmol, 1.2 equiv) was added dropwise at −78 °C. The reaction mixture was stirred at −78 °C for 1 h. Methyl chloroformate (0.08 mL, 1.0 mmol, 3 equiv) was added dropwise. The reaction mixture was stirred at −78 °C for another 1.5 h. The reaction mixture was quenched with NH_4_Cl and extracted with EtOAc. The combined organic layers were washed with brine, dried (Na_2_SO_4_), and concentrated; and the residue was purified by flash column chromatography (30:1 to 10:1 Hex:EtOAc) to give **30** (21.3 mg, 0.0615 mmol, 18%) as a pale-yellow oil. ^1^H-NMR (500 MHz, CDCl_3_): δ 7.72 (nfod, *J_app_* = 8.4 Hz, 2H), 7.31 (nfod, *J_app_* = 8.3 Hz, 2H), 5.17 (m, 1H), 5.09 (m, 1H), 4.27 (s, 2H), 4.26 (s, 2H), 3.73 (s, 3H), 2.41 (s, 3H), and 1.71 (dd, *J* = 1.3, 1.4 Hz, 3H). ^13^C-NMR{^1^H} (126 MHz, CDCl_3_): δ 153.2, 144.3, 135.0, 129.9, 128.0, 125.9, 122.9, 87.7, 80.6, 80.0, 77.1, 52.9, 37.7, 36.5, 23.1, and 21.7. IR (neat): ν_max_ 2954 (C_sp3_-H), 2922 (C_sp3_-H), 2847 (C_sp3_-H), 2242 (C≡C), and 1717 (C=O) cm^−1^. HRMS (ESI-TOF): Calcd for C_18_H_20_NO_4_S^+^ [M + H]^+^ 346.1108; found 346.1094 (100%).

#### 3.1.3. Synthesis of Products of the TDDA Reactions

6-Methyl-4-(prop-1-en-2-yl)-2-tosylisoindoline (**16**) and 6-methyl-4-(propan-2-ylidene)-2-tosyl-4,5-dihydro-2H-isoindole (**17**). In a 10 mL culture tube, equipped with a magnetic stir bar, **14** (21 mg, 0.064 mmol) was added. 1,2-DCE (2 mL) was then added. The reaction mixture was heated at 130 °C for 16 h. The reaction mixture was concentrated and purified by flash column chromatography (30:1 Hex:EtOAc) to give, in order of elution, a small amount of the pyrrole derivative **17**, contaminated with a small amount of co-eluting remaining starting material **14**, and the major product, **16** (6.3 mg, 0.019 mmol, 30%) as a white crystalline solid. The ratio of **17**:**16** in this experiment was ca. 1:2 (analysis of the ^1^H NMR spectrum of the crude product mixture prior to chromatographic purification). The pyrrole derivative showed evidence of slowly giving rise to several new compounds over time and handling (TLC and NMR). The experiment was repeated on a larger scale [50 mg **14** in 1.5 mL of DCE in the presence of added BHT (0.1 equiv); 130 °C, ca. 16 h]. The ratio of products **17**:**16** was now 1:6 (crude NMR). MPLC (5:1 Hex:EtOAc) provided a purer sample of the pyrrole derivative **17** (10 mg, 10%, corrected for residual EtOAc and hexanes, which were purposely left to minimize loss of the somewhat volatile product; this is the sample for which the characterization data were recorded) and the isoindoline **16** (30 mg, 60%), respectively. **16:** mp: 138–142 °C. ^1^H-NMR (500 MHz, CDCl_3_): δ 7.76 (nfod, *J_app_* = 8.2 Hz, 2H), 7.31 (nfod, *J_app_* = 8.0 Hz, 2H), 6.95 (dq, *J* = 1.6, 0.8 Hz, 1H), 6.88 (br dq, *J* = 1.6, 0.8 Hz, 1H), 5.18 (dq, *J* = 1.5, 1.5 Hz, 1H), 4.92 (dq, *J* = 1.4, 1.0 Hz, 1H), 4.60 (nfom, 2H), 4.57 (nfom, 2H), 2.40 (s, 3H), 2.30 (dd, *J* = 0.8, 0.8 Hz, 3H), and 2.04 (dd, *J* = 1.5, 1.0 Hz, 3H). ^13^C-NMR{^1^H} (126 MHz, CDCl_3_): δ 143.8, 143.1, 138.4, 138.0, 136.9, 133.8, 130.4, 129.9, 127.7, 127.2, 121.9, 115.5, 53.70, 53.69, 23.4, 21.6, and 21.3. IR (neat): ν_max_ 3016 (C_sp2_-H), 2920 (C_sp3_-H), and 2847 (C_sp3_-H) cm^−1^. HRMS (ESI-TOF): Calcd for C_19_H_22_NO_2_S^+^ [M + H]^+^ 328.1366; found 328.1351 (100%). **17:** ^1^H-NMR (500 MHz, CDCl_3_): δ 7.72 (nfod, *J_app_* = 8.3 Hz, 2H), 7.25 (nfod, *J_app_* = 8.2 Hz, 2H), 7.03 (br d, *J* = 2.3 Hz, 1H), 6.82 (d, *J* = 2.1 Hz, 1H), 6.07 (tqd, simulated as *J* = 2.1, 1.3, 0.8 Hz, 1H), 3.05–2.97 (m, Σ*J*s = ~14 Hz, 2H), 2.38 (t, *J* = 0.8 Hz, 3H), 1.93 (br t, *J* = 2.2 Hz, 3H), 1.82 (br m, 3H), and 1.81 (dt, *J* = 1.3, 1.3 Hz, 3H). ^13^C-NMR{^1^H} (126 MHz, CDCl_3_): δ 144.7, 136.6, 136.3, 130.0, 129.4, 126.84, 126.78, 124.0, 120.6, 117.8, 114.0, 113.3, 35.7, 24.1, 23.4, 22.3, and 21.7. HRMS (ESI-TOF): Calcd for C_19_H_22_NO_2_S^+^ [M + H]^+^ 328.1366; found 328.1357. This compound showed signs of oxidative decomposition upon handling and storage, and the HRMS data showed ions consistent with such processes (M + H^+^–H_2_, M + H^+^ + O, M + H^+^ – H_2_ + O).

6-Methyl-4-(prop-1-en-2-yl)-2-tosylisoindoline-7-d (**16-d**) and 6-Methyl-4-(propan-2-ylidene)-2-tosyl-4,5-dihydro-2H-isoindole-7-d (**17-d**). In a 10 mL culture tube, equipped with a magnetic stir bar, **14** (21 mg, 0.064 mmol) was added. 1,2-DCE (1.8 mL) and CD_3_OD (0.2 mL) were then added. The reaction mixture was heated at 130 °C for 16 h. The reaction mixture was concentrated and purified by flash column chromatography (30:1 Hex:EtOAc) to give **16-d** (6.12 mg, 0.019 mmol, 29%) as a white crystalline solid. The ratio of **17-d**:**16-d** in this experiment was ca. 1:2 (analysis of the ^1^H NMR spectrum of the crude product mixture prior to chromatographic purification). **16-d**: mp: 136–140 °C. ^1^H-NMR (500 MHz, CDCl_3_): δ 7.76 (nfod, *J_app_* = 8.3 Hz, 2H), 7.31 (nfod, *J_app_* = 8.1 Hz, 2H), 6.95 (q, *J* = 0.8 Hz, 1H), 6.88 (s, 0.09H), 5.18 (dq, *J* = 1.6, 1.6 Hz, 1H), 4.92 (dq, *J* = 1.4, 1.0 Hz, 1H), 4.60 (nfom 2H), 4.57 (nfom 2H), 2.40 (s, 3H), 2.30 (d, *J* = 0.8 Hz, 3H), and 2.04 (dd, *J* = 1.5, 1.1 Hz, 3H). ^13^C-NMR{^1^H} (126 MHz, CDCl_3_): δ 143.7, 143.1, 138.4, 137.9, 136.8, 133.8, 130.5, 129.9, 127.8, 127.2, 115.5, 53.71, 53.68, 23.4, 21.6, and 21.3. (No resonance was observed at 122.0 ppm for the now-deuterated carbon.) IR (neat): ν_max_ 3016 (C_sp2_-H), 2919 (C_sp3_-H), and 2848 (C_sp3_-H) cm^−1^. HRMS (ESI-TOF): Calcd for C_19_H_21_DNO_2_S^+^ [M + H]^+^ 329.1429; found 329.1414 (100%). **17-d**: ^1^H-NMR (500 MHz, CDCl_3_): δ 7.72 (nfod, *J_app_* = 8.3 Hz, 2H), 7.25 (nfod, *J_app_* = 8.2 Hz, 2H), 7.04 (br d, *J* = 2.1 Hz, 1H), 6.82 (d, *J* = 2.1 Hz, 1H), 6.07 (m, 0.1H), 3.00–2.97 (m, Σ*J*s = ~14 Hz, 2H), 2.38 (t, *J* = 0.9 Hz, 3H), 1.93 (td, *J* = 2.0, 0.9 Hz, 3H), 1.82 (br m, 3H), and 1.81 (t, *J* = 1.2 Hz, 3H).

Methyl 6-Methyl-2-tosylisoindoline-4-carboxylate (**33**). In a 10 mL culture tube, equipped with a magnetic stir bar, **30** (30 mg, 0.087 mmol) was added. 1,2-DCE (2 mL) was added. The reaction mixture was heated to 130 °C for 16 h. The reaction mixture was concentrated and purified by flash column chromatography (10:1 Hex:EtOAc) to give **33** (12.61 mg, 0.037 mmol, 42%) as a white crystalline solid. mp: 175–180 °C. ^1^H-NMR (500 MHz, CDCl_3_): δ 7.78 (nfod, *J_app_* = 8.0 Hz, 2H), 7.72 (s, 1H), 7.30 (nfod, *J_app_* = 7.9 Hz, 2H), 7.16 (s, 1H), 4.87 (s, 2H), 4.60 (s, 2H), 3.89 (s, 3H), 2.39 (s, 3H), and 2.35 (s, 3H). ^13^C-NMR{^1^H} (126 MHz, CDCl_3_): δ 166.4, 143.8, 138.3, 137.9, 135.7, 134.0, 130.2, 130.0, 127.73, 127.72, 125.2, 54.9, 53.2, 52.3, 21.6, and 21.2. IR (neat): ν_max_ 2952 (C_sp3_-H), 2923 (C_sp3_-H), 2856 (C_sp3_-H), and 1721(C=O) cm^−1^. HRMS (ESI-TOF): Calcd for C_18_H_20_NO_4_S^+^ [M + H]^+^ 346.1108; found 346.1094 (100%).

Methyl 6-Methyl-2-tosylisoindoline-4-carboxylate-7-*d* (**33-d**). In a 10 mL culture tube, equipped with a magnetic stir bar, **30** (21 mg, 0.064 mmol) was added. 1,2-DCE (1.8 mL) and CD_3_OD (0.2 mL) were then added. The reaction mixture was heated at 130 °C for 16 h. The reaction mixture was concentrated and purified by flash column chromatography (10:1 Hex:EtOAc) to give methyl 6-methyl-2-tosylisoindoline-4-carboxylate-7-*d* (**33-d**, 5.90 mg, 0.017 mmol, 27%) as a white crystalline solid: mp: 186–190 °C. ^1^H-NMR (500 MHz, CDCl_3_): δ 7.78 (nfod, *J_app_* = 8.3 Hz, 2H, *H2*), 7.72 (q, *J* = 0.8 Hz, 1H, *H5′*), 7.30 (nfod, *J_app_* = 8.2 Hz, 2H, *H3*), 7.16 (s, 0.1H, H7′), 4.87 (nfot, *J_app_* = 2.1 Hz, 2H, *H2′*), 4.60 (nfot, *J_app_* = 2.2 Hz, 2H, *H9′*), 3.89 (s, 3H, CO_2_C*H_3_*), 2.39 (s, 3H, C4C*H_3_*), and 2.35 (d, *J* = 0.9 Hz, 3H, C6′C*H_3_*). ^13^C-NMR{^1^H} (126 MHz, CDCl_3_): δ 166.4, 143.8, 138.2, 137.8, 135.7, 134.0, 130.2, 130.0, 127.74, 125.3, 54.9, 53.2, 52.3, 21.7, and 21.1. (No resonance was observed at 127.72, the largely deuterated C7′). IR (neat): ν_max_ 2952 (C_sp3_-H), 2921 (C_sp3_-H), 2850 (C_sp3_-H), and 1721(C=O) cm^−1^. HRMS (ESI-TOF): [M + H]^+^ Calcd for C_18_H_19_DNO_4_S^+^ 347.1170; Found 347.1155 (100%).

## 4. Conclusions

The symmetrical bis-enyne **14** represents a rare type of substrate used for study of a thermally driven tetradehydro-Diels–Alder reaction. As such, the initially produced strained 1,2,4-cyclohexatriene intermediate **15** uniquely had an alkene (a 2-propenyl group) attached to C5. Heating **14** gave rise to not only the expected aromatic benzenoid product, **16**, but a second, unusual isomeric side product, **17**, whose structure was not immediately apparent. After detailed NMR analyses, it was concluded that **17** was a pyrrole derivative. Its formation is best rationalized by a [1,5]-hydrogen atom migration within **15** (or **21**; cf. Figure 3). Deuteration studies using CD_3_OD or (CH_3_OD) showed that the presence of a protic additive was involved in forming both products **16** and **17**. When the more acidic protic additive BHT was used, formation of the pyrrole side product **17** was completely suppressed, suggesting that the acidity of the OH additive played an important role in determining the fate of the 1,2,4-cyclohexatriene derivative **15**.

## Data Availability

Data are contained within the article or its Supplementary Materials.

## Supplementary Materials

The following supporting information can be downloaded at https://www.mdpi.com/article/10.3390/molecules30122610/s1: a PDF containing copies of ^1^H and ^13^C NMR spectra for all new compounds and the data resulting from the DP4+ analysis; a zip file of the Gaussian out files used in the DP4+ analysis; and a zip file of Mnova files containing raw NMR fid data of all new structures.

## Abbreviations

The following abbreviations are used in this manuscript:

ATR	attenuated total reflectance
BHT	butylated hydroxytoluene
DCE	dichloroethane
DCM	dichloromethane
ESI	electrospray ionization
EtOAc	ethyl acetate
GCMS	gas chromatography–mass spectrometry
Hex	hexanes
IR	infrared
mp	melting point
MPLC	medium pressure liquid chromatography
NMR	nuclear magnetic resonance
NOEs	nuclear Overhauser effects
NOESY	nuclear Overhauser effect spectroscopy
NTs	*N*-tosyl
SI	supporting information
TDDA	tetradehydro-Diels–Alder
THF	tetrahydrofuran
TLC	thin layer chromatography
