# Supplementary material for: A Case of Competitive Aromatization vs. Sigmatropic [1,5]-Hydrogen Atom Migration in a 1,2,4-Cyclohexatriene Intermediate Derived from a Bis-Enyne Cyclization
Source: Molecules. 2025 Jun 16;30(12):2610. doi: 10.3390/molecules30122610 (PMC12195621; doi:10.3390/molecules30122610)
Supplement: Supplementary file 1 [file molecules-30-02610-s001.zip › All SI Data/SI Revision Merged Text + NMR.pdf.zip!/SI Revision Merged Text + NMR.pdf]

Supporting Information

for

A Case of Competitive Aromatization vs. Sigmatropic [1,5]-Hydrogen Atom Migration in a 1,2,4-Cyclohexatriene Intermediate Derived from a bis-Enyne Cyclization

Rong Tang, Qian Xu, and Thomas R. Hoye*

Department of Chemistry, University of Minnesota, 207 Pleasant St. SE, Minneapolis, Minnesota 55455

Email: hoye@umn.edu

Table of Contents

I. General Experimental Protocols S2

II. Experimental Procedures and Characterization Data for New Compounds S3–S11

New compounds **S2**, **S4** not in the manuscript S3–S4

New compounds **14**, **16**, **16-d**, **17**, **17-d**, **30**, **33**, **33-d** in the manuscript S5–S11

III. DP4+ NMR analysis of structures 17 (isomer 1) and S5 (isomer 2) S12–S14

IV. Supporting Information References S15

V. Copies of NMR Spectra S15

S2 ^{1}H/^{13}C S16–S17

S4 ^{1}H/^{13}C S18–S19

14 ^{1}H/^{13}C S20–S21

16 ^{1}H/^{13}C/NOESY/HSQC/HMBC S22–S26

16-d ^{1}H/^{13}C S27–S28

17 ^{1}H/^{13}C/NOESY/HSQC/HMBC S29–S33

17-d ^{1}H (from crude mixture containing **16-d**) S34

30 ^{1}H/^{13}C S35–S36

33 ^{1}H/^{13}C S37–S38

33-d ^{1}H/^{13}C S39–S40

I. General Experimental Protocols

^{13}C and ^{1}H NMR spectra were obtained using a Bruker HD-500 spectrometer. All spectra were recorded in $CDCl_3$. The reported proton chemical shifts are referenced to $CHCl_3$ in (d = 7.26 ppm) in $CDCl_3$. Resonances in the line listings of ^{1}H NMR spectral data are given in this format: chemical shift (d, ppm) [multiplicity, coupling constant(s) J (in Hz), integral value (to the nearest integer)]. First-order coupling constants have been extracted using methods we have described elsewhere.[1],[2] A nfom, nfod, or nfot refers to a non-first order multiplet, doublet, or triplet in a ^{1}H NMR spectrum. Coupling constants for these resonances are given as apparent values (J_{app}); (e.g., the observed spacing between the two most intense lines in a nfod is the value of $J_o + J_p$ and not the true doublet coupling constant. The reported carbon chemical shifts in ^{13}C spectra are referenced to $CDCl_3$ (d = 77.16 ppm).

Infrared spectra were recorded using a using a Bruker Alpha II Spectrometer. The data were collected in attenuated total reflectance (ATR) mode. Samples were measured as thin films that were deposited onto a diamond ATR window by evaporative loss of solvent, often $CDCl_3$. Absorption maxima are given as cm^{-1}.

High-resolution mass spectrometry (HRMS) data were collected in the ESI mode with a Thermo Orbitrap Velos instrument having mass accuracy of ≤3 ppm. Pierce™ LTQ was used as an external calibrant. Samples were injected directly into the ion source.

Melting points were measured on a Köfler hot-stage and polarizing microscope and are uncorrected.

New substances were often purified by **medium pressure liquid chromatography** (MPLC) at 25-200 psi. Silica gel columns (hand-packed, normal-phase, Teledyne RediSep Rf Gold®, 20-40 µm, 60 Å pore size) were used. The unit was assembled from a Waters HPLC pump (model 510) affixed to a Waters differential refractive index detector (R401).

Preparative flash column chromatography was done on columns hand-packed with Agela silica gel (230-400 mesh).

Thin layer chromatography (TLC) was carried out on silica-gel coated plates (plastic-backed), visualized by UV light and/or by staining with a solution of potassium permanganate.

Reaction temperatures refer to the temperature of a pre-equilibrated and external heating oil bath. Any reaction performed at a temperature higher than that of the reaction solvent's boiling point were carried out in a threaded culture tube, which was closed with an inert, Teflon®-lined screw-cap.

II. Experimental Procedures and Characterization Data for New Compounds

N,*N*-*bis*(4-Hydroxy-4-methylpent-2-yn-1-yl)-4-methylbenzenesulfonamide (S2)

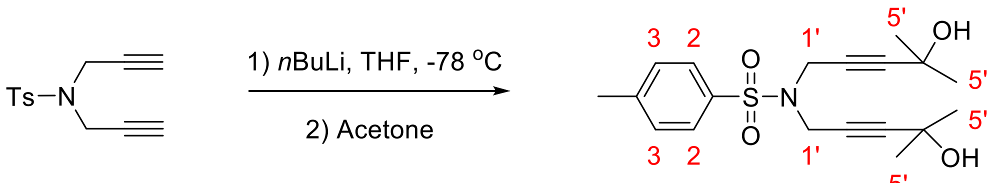

S1 ***S2***

In a 100 mL round-bottomed flask equipped with a magnetic stir bar, 4-methyl-*N*,*N*-di(prop-2-yn-1-yl)benzenesulfonamide (**S1**, 2.3 g, 9.4 mmol, 1 equiv) was added. THF (47 mL) was added at -78 °C under an atmosphere of N_2. *n*-BuLi (7.9 mL, 2.5 M in Hexanes, 2.1 equiv) was added dropwise. After 1 h of stirring at -78 °C, acetone (2.1 mL, 28 mmol, 3 equiv) was added dropwise. The reaction mixture was stirred at -78 °C for another 1 h. The reaction was quenched with saturated NH_4Cl aqueous solution. THF was removed under vacuum and the aqueous layer was extracted with DCM, washed with brine, dried (Na_2SO4), concentrated, and purified by flash column chromatography (10:1 to 1:1 Hex:EtOAc) to give **S2** (2.9 g, 8.0 mmol, 85%) as a white solid.

^{1}H-NMR (500 MHz, $CDCl_3$): δ 7.76 (nfod, J_{app} = 8.3 Hz, 2H, *H2*), 7.33 (nfod, J_{app} = 8.0 Hz, 2H, *H3*), 4.17 (s, 4H, *H1′*), 2.43 (s, 3H, ArC*H*$_3$), 1.89 (s, 2H, O*H*), and 1.37 (s, 12H, *H5′*).

^{13}C-NMR{^{1}H} (126 MHz, $CDCl_3$): δ 144.0, 136.1, 129.7, 128.3, 90.5, 74.9, 65.1, 37.1, 31.2, and 21.6.

IR (neat): 3524 (br O-H), 3265 (br O-H), 2981 (CH_3), and 2924 (CH_2) cm^{-1}.

HRMS (ESI-TOF): $[M+H]^+$ Calcd for $C_{19}H_{26}NO_4S^+$ 364.1577, found 364.1560 (9%).

TLC: R_f 0.4 (1:1 Hex:EtOAc).

mp: 98–100 °C.

4-Methyl-*N*-(4-methylpent-4-en-2-yn-1-yl)-*N*-(prop-2-yn-1-yl)benzenesulfonamide (S4)

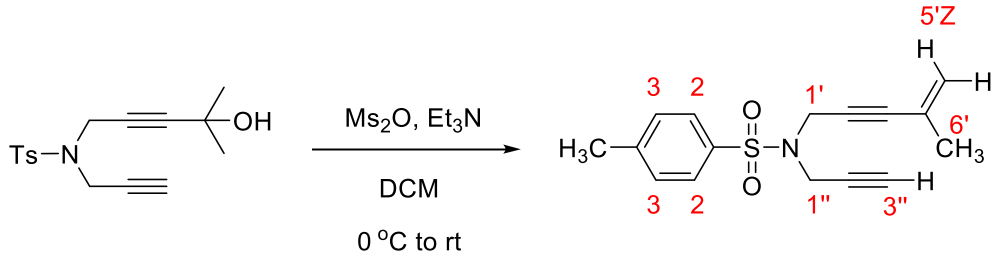

S3 ***S4***

In a culture tube, equipped with a magnetic stir bar, *N*-(4-hydroxy-4-methylpent-2-yn-1-yl)-4-methyl-*N*-(prop-2-yn-1-yl)benzenesulfonamide (**S3**, 305 mg, 1 mmol, 1 equiv) was added. DCM (5.9 mL) was added under an atmosphere of N_2. Triethylamine (2.4 mL, 17.6 mmol, 17.6 equiv) was added at 0 °C. Methanesulfonic anhydride (1.5 g, 8.8 mmol, 8.8 equiv) was added dropwise. The reaction mixture was stirred at rt for 1 h and then poured into ice-cold concentrated aqueous HCl (2 mL). The aqueous layer was extracted with DCM. The combined organic layers were washed with brine, dried (Na_2SO_4), filtered, concentrated, and purified by MPLC (15:1 Hex:EtOAc) to give **S4** (162.88 mg, 0.56 mmol, 57%) as a pale-yellow oil.

^{1}H-NMR (500 MHz, $CDCl_3$): δ 7.72 (nfod, J_{app} = 8.4 Hz, 2H, *H2*), 7.29 (nfod, J_{app} = 8.1 Hz, 2H, *H3*), 5.15 (dq, *J* = 1.6, 1.6 Hz, 1H, *H5'E* or *H5'Z*), 5.07 (dq, *J* = 1.8, 1.0 Hz, 1H, *H5'Z* or *H5'E*), 4.29 (s, 2H, *H1'*), 4.13 (dd, *J* = 2.5, 0.7 Hz, 2H, *H1"*), 2.41 (t, *J* = 0.9 Hz, 3H, ArC*H3*), 2.16 (t, *J* = 2.5 Hz, 1H, *H3"*), and 1.71 (dd, *J* = 1.6, 1.1 Hz, 3H, *H6'*).

^{13}C{^{1}H} (126 MHz, $CDCl_3$): δ 144.0, 135.4, 129.7, 128.1, 126.0, 122.5, 87.2, 80.4, 76.6, 74.0, 37.1, 36.5, 23.2, and 21.7.

IR (neat): 3278 (C_{sp}-H), 2982 (H_2C_{sp3}-H), 2927 (RHC_{sp3}-H), and 1597 (C=C) cm^{-1}

HRMS (ESI-TOF): $[M+H]^+$ Calcd for $C_{16}H_{18}NO_2S^+$ 288.1053; Found 288.1060 (100%).

TLC: R_f 0.19 (10:1 Hex:EtOAc).

4-Methyl-*N*,*N*-*bis*(4-methylpent-4-en-2-yn-1-yl)benzenesulfonamide (14)

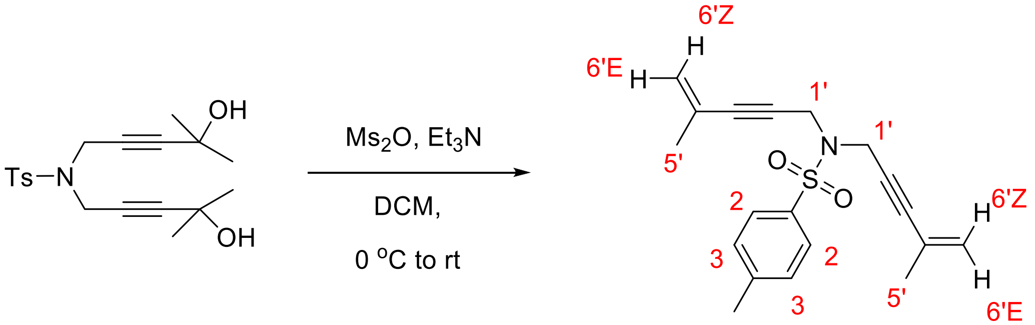

S2 ***14***

In a 100 mL round-bottomed flask, equipped with a magnetic stir bar, **S2** (790 mg, 2.2 mmol, 1 equiv) was added. DCM (13 mL) was added under a N_2 atmosphere. To this solution, triethylamine (10.6 mL, 76 mmol, 35 equiv) was added at 0 °C. Methanesulfonic anhydride (6.7 g, 38 mmol, 18 equiv) dissolved in DCM (26 mL) was added dropwise. The reaction mixture was stirred at rt for 2.5 h. The reaction mixture was quenched with water and extracted with DCM. The combined organic layers were washed with brine, dried (Na_2SO_4), filtered, and concentrated; and the residue was purified by flash column chromatography (10:1 Hex:EtOAc) to give **14** (511 mg, 1.6 mmol, 72%) as a pale-yellow oil.

^{1}H-NMR (500 MHz, $CDCl_3$): δ 7.76 (nfod, J_{app} = 8.0 Hz, 2H, *H2*), 7.31 (nfod, J_{app} = 8.2 Hz, 2H, *H3*), 5.19 (br s, 2H, *H6'E* or *H6'Z*), 5.13 (br s, 2H, *H6'E* or *H6'Z*), 4.29 (s, 4H, *H1'*), 2.42 (s, 3H, ArCH_3), and 1.76 (br s, *J* = 1.3 Hz, 6H, *H5'*).

^{13}C-NMR{^{1}H} (126 MHz, $CDCl_3$): δ 143.8, 135.6, 129.7, 128.1, 126.1, 122.5, 87.0, 80.8, 37.3, 23.2, and 21.6.

IR (neat): 3003 (C_{sp2}-H), 2925 (H_2C_{sp3}-H), and 1598 (C=C) cm^{-1}.

HRMS (ESI-TOF): $[M+H]^+$ Calcd for $C_{19}H_{22}NO_2S^+$ 328.1366; Found 328.1353 (100%).

TLC: R_f 0.4 (10:1 Hex:EtOAc).

6-Methyl-4-(prop-1-en-2-yl)-2-tosylisoindoline (16)

and

6-Methyl-4-(propan-2-ylidene)-2-tosyl-4,5-dihydro-2H-isoindole (17)

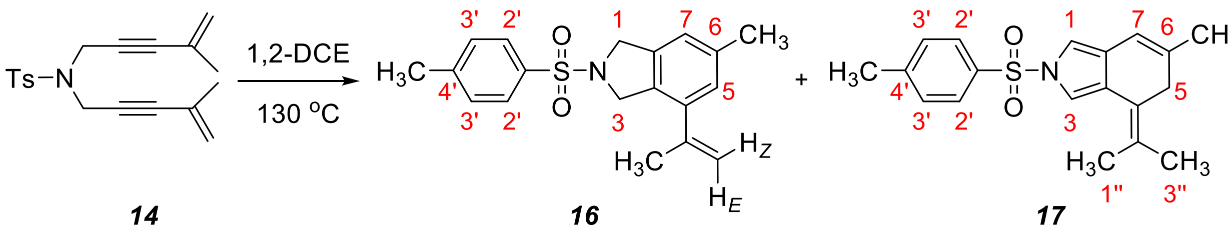

14 ***16*** ***17***

In a 10 mL culture tube, equipped with a magnetic stir bar, **14** (21 mg, 0.064 mmol) was added. 1,2-DCE (2 mL) was added. The reaction mixture was heated at 130 °C for 16 h. The reaction mixture was concentrated and purified by flash column chromatography (30:1 Hex:EtOAc) to give, in order of elution, a small amount of the pyrrole derivative **17**, contaminated with a small amount of co-eluting remaining starting material **14**, and the major product, 6-methyl-4-(prop-1-en-2-yl)-2-tosylisoindoline (**16**, 6.3 mg, 0.019 mmol, 30%) as a white crystalline solid. The ratio of **16**:**17** in this experiment was ca. 1:2 (analysis of the ^{1}H NMR spectrum of the crude product mixture prior to chromatographic purification). The pyrrole derivative showed evidence of slowly giving rise to several new compounds over time and handling (TLC and NMR).

The experiment was repeated on a larger scale [50 mg **14** in 1.5 mL of DCE in the presence of added BHT (0.1 equiv); 130 °C, ca. 16 h]. The ratio of products **17**:**16** was now 1:6 (crude NMR). MPLC (5:1 Hex:EtOAc) provided a purer sample of the pyrrole derivative **17** (10 mg, 10%, corrected for residual EtOAc and hexanes, which were purposely left to minimize loss of the volatile product; this is the sample for with which the characterization data were recorded) and the isoindoline **16** (30 mg, 60%) respectively.

16 (major product)

^{1}H-NMR (500 MHz, $CDCl_3$): δ 7.76 (nfod, J_{app} = 8.2 Hz, 2H, *H2'*), 7.31 (nfod, J_{app} = 8.0 Hz, 2H, *H3'*), 6.95 (dq, *J* = 1.6, 0.8 Hz, 1H, *H5*), 6.88 (br dq, *J* = 1.6, 0.8 Hz, 1H, *H7*), 5.18 (dq, *J* = 1.5, 1.5 Hz, 1H, H_E), 4.92 (dq, *J* = 1.4, 1.0 Hz, 1H, H_Z), 4.60 (nfom, 2H, *H3*), 4.57 (nfom, 2H, *H1*), 2.40 (s, 3H, C4'CH_3), 2.30 (dd, *J* = 0.8, 0.8 Hz, 3H, C6CH_3), and 2.04 (dd, *J* = 1.5, 1.0 Hz, 3H, H_2C=CCH_3).

NOESY (larger cis-allylic coupling constant for H_E to Me (1.5 Hz) than trans-J H_Z to Me (1.0 Hz)

^{13}C-NMR{^{1}H} (126 MHz, $CDCl_3$): δ 143.8 (*C4'*), 143.1 (*C4*), 138.4 (Me*C*=CH_2), 138.0 (*C6*), 136.9 (*C3a* or *C7a*), 133.8 (*C1'*), 130.4 (*C3a* or *C7a*), 129.9 (*C3'*), 127.7 (*C2'*), 127.2 (*C5*), 121.9 (*C7*), 115.5 (=*C*H_2), 53.70 (*C1* or *C3*), 53.69 (*C1* or *C3*), 23.4 (=C*C*H_3), 21.6 (C4'*C*H_3), and 21.3 (C6*C*H_3).

HSQC HMBC

IR (neat): 3016 (C_{sp2}-H), 2920 (C_{sp3}-H), and 2847 (C_{sp3}-H) cm^{-1}.

HRMS (ESI-TOF): $[M+H]^+$ Calcd for $C_{19}H_{22}NO_2S^+$ 328.1366; Found 328.1351 (100%).

TLC: R_f 0.2 (10:1 Hex:EtOAc).

mp: 138–142 °C.

17 (minor product, the pyrrole derivative)

^{1}H-NMR (500 MHz, $CDCl_3$): δ 7.72 (nfod, J_{app} = 8.3 Hz, 2H, *H2'*), 7.25 (nfod, J_{app} = 8.2 Hz, 2H, *H3'*), 7.03 (br d, *J* = 2.3 Hz, 1H, *H3*), 6.82 (d, *J* = 2.1 Hz, 1H, *H1*), 6.07 (tqd, simulated as *J* = 2.1, 1.3, 0.8 Hz, 1H, *H7*), 3.05–2.97 (m, Σ*J*s = ~14 Hz, 2H, *H5*), 2.38 (t, *J* = 0.8 Hz, 3H, C4'CH_3), 1.93 (br t, *J* = 2.2 Hz, 3H, *H1''*), 1.82 (br m, 3H, *H3''*), and 1.81 (dt, *J* = 1.3, 1.3 Hz, 3H, C6CH_3).

NOESY

^{13}C-NMR{^{1}H} (126 MHz, $CDCl_3$): δ 144.7 (*C4'*), 136.6 (*C6*), 136.3 (*C1'*), 130.0 (*C3'*), 129.4 (*C2''*), 126.84 (*C2'*), 126.78 (*C3a* or *C7a*), 124.0 (*C3a* or *C7a*), 120.6 (*C4*), 117.8 (*C3*), 114.0 (*C7*), 113.3 (*C1*), 35.7 (*C5*), 24.1 (*C1''*), 23.4 (C6*C*H_3), 22.3 (*C3''*), and 21.7 (C4'*C*H_3).

HSQC HMBC

HRMS (ESI-TOF): $[M+H]^+$ Calcd for $C_{19}H_{22}NO_2S^+$ 328.1366; Found 328.1357.

6-Methyl-4-(prop-1-en-2-yl)-2-tosylisoindoline-7-d (16-d)

and

6-Methyl-4-(propan-2-ylidene)-2-tosyl-4,5-dihydro-2H-isoindole-7-d (17-d)

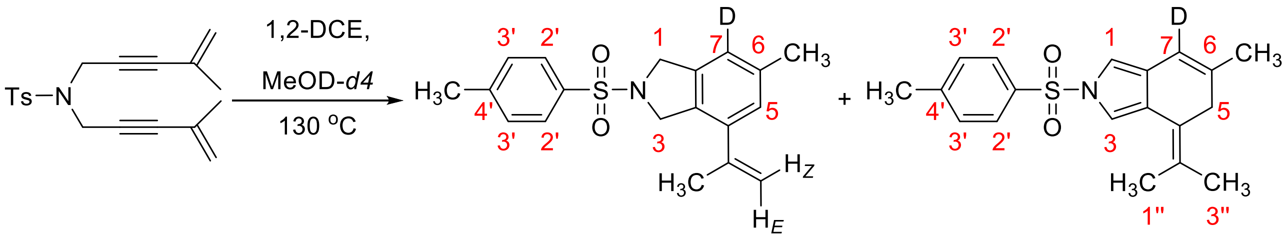

14 ***16-d*** ***17-d***

In a 10 mL culture tube, equipped with a magnetic stir bar, **14** (21 mg, 0.064 mmol) was added. 1,2-DCE (1.8 mL) and CD_3OD (0.2 mL) were added. The reaction mixture was heated at 130 °C for 16 h. The reaction mixture was concentrated and purified by flash column chromatography (30:1 Hex:EtOAc) to give 6-methyl-4-(prop-1-en-2-yl)-2-tosylisoindoline-7-d (**16-d**, 6.12 mg, 0.019 mmol, 29%) as a white crystalline solid. The ratio of **17-d**:**16-d** in this experiment was ca. 1:2 (analysis of the ^{1}H NMR spectrum of the crude product mixture prior to chromatographic purification).

16-d (major product)

^{1}H-NMR (500 MHz, $CDCl_3$): δ 7.76 (nfod, J_{app} = 8.3 Hz, 2H, *H2'*), 7.31 (nfod, J_{app} = 8.1 Hz, 2H, *H3'*), 6.95 (q, *J* = 0.8 Hz,1H, *H5*), 6.88 (s, 0.09H, *H7*), 5.18 (dq, *J* = 1.6, 1.6 Hz, 1H, H_E), 4.92 (dq, *J* = 1.4, 1.0 Hz, 1H, H_Z), 4.60 (nfom 2H, *H3*), 4.57 (nfom 2H, *H1*), 2.40 (s, 3H, C4'CH_3), 2.30 (d, *J* = 0.8 Hz, 3H, C6CH_3), and 2.04 (dd, *J* = 1.5, 1.1 Hz, 3H, H_2C=CCH_3).

^{13}C-NMR{^{1}H} (126 MHz, $CDCl_3$): δ 143.7, 143.1, 138.4, 137.9, 136.8, 133.8, 130.5, 129.9, 127.8, 127.2, 115.5, 53.71, 53.68, 23.4, 21.6, and 21.3. (no resonance observed at 122.0 ppm for the now-deuterated carbon)

IR (neat): 3016 (C_{sp2}-H), 2919 (C_{sp3}-H), and 2848 (C_{sp3}-H) cm^{-1}.

HRMS (ESI-TOF): $[M+H]^+$ Calcd for $C_{19}H_{21}DNO_2S^+$ 329.1429; Found 329.1414 (100%).

TLC: R_f 0.2 (10:1 Hex:EtOAc).

mp: 136–140 °C .

17-d (minor product, the pyrrole derivative)

^{1}H-NMR (500 MHz, $CDCl_3$): δ 7.72 (nfod, J_{app} = 8.3 Hz, 2H, *H2'*), 7.25 (nfod, J_{app} = 8.2 Hz, 2H, *H3'*), 7.04 (br d, *J* = 2.1 Hz, 1H, *H3*), 6.82 (d, *J* = 2.1 Hz, 1H, *H1*), 6.07 (m, 0.1H, *H7*), 3.00-2.97 (m, Σ*J*s = ~14 Hz, 2H, *H5*), 2.38 (t, *J* = 0.9 Hz, 3H, C4'CH_3), 1.93 (td, *J* = 2.0, 0.9 Hz, 3H, *H1''*), 1.82 (br m, 3H, *H3''*), and 1.81 (t, *J* = 1.2 Hz, 3H, C6CH_3).

Methyl 4-{[4-Methyl-*N*-(4-methylpent-4-en-2-yn-1-yl)phenyl]sulfonamido}but-2-ynoate (30)

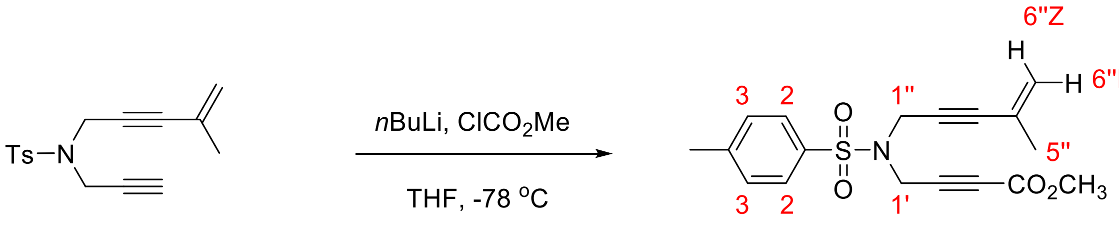

S4 ***30***

In a 10 mL round-bottomed flask, equipped with a magnetic stir bar, **S4** (100 mg, 0.35 mmol, 1 equiv) was added. THF (3.5 mL) was added under a N_2 atmosphere. *n*BuLi (0.17 mL, 0.42 mmol, 1.2 equiv) was added dropwise at -78 °C. The reaction mixture was stirred at -78 °C for 1 h. Methyl chloroformate (0.08 mL, 1.0 mmol, 3 equiv) was added dropwise. The reaction mixture was stirred at -78 °C for another 1.5 h. The reaction mixture was quenched with NH_4Cl and extracted with EtOAc. The combined organic layers were washed with brine, dried (Na_2SO_4), and concentrated; and the residue was purified by flash column chromatography (30:1 to 10:1 Hex:EtOAc) to give **30** (21.3 mg, 0.0615 mmol, 18%) as a pale-yellow oil.

^{1}H-NMR (500 MHz, $CDCl_3$): δ 7.72 (nfod, J_{app} = 8.4 Hz, 2H, *H2*), 7.31 (nfod, J_{app} = 8.3 Hz, 2H, *H3*), 5.17 (m, 1H, *H6"E* or *H6"Z*), 5.09 (m, 1H, *H6"E* or *H6"Z*), 4.27 (s, 2H, *H1'*), 4.26 (s, 2H, *H1"*), 3.73 (s, 3H, CO_2CH_3), 2.41 (s, 3H, ArCH_3), and 1.71 (dd, *J* = 1.3, 1.4 Hz, 3H, *H5"*).

^{13}C-NMR{^{1}H} (126 MHz, $CDCl_3$): δ 153.2, 144.3, 135.0, 129.9, 128.0, 125.9, 122.9, 87.7, 80.6, 80.0, 77.1, 52.9, 37.7, 36.5, 23.1, and 21.7.

IR (neat): 2954 (C_{sp3}-H), 2922 (C_{sp3}-H), 2847 (C_{sp3}-H), 2242 (C≡C), and 1717 (C=O) cm^{-1}.

HRMS (ESI-TOF): $[M+H]^+$ Calcd for $C_{18}H_{20}NO_4S^+$ 346.1108; Found 346.1094 (100%).

TLC: R_f 0.4 (10:1 Hex:EtOAc).

Methyl 6-Methyl-2-tosylisoindoline-4-carboxylate (33)

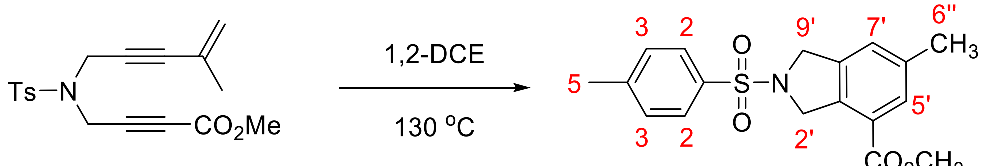

30 ***33***

In a 10 mL culture tube, equipped with a magnetic stir bar, **30** (30 mg, 0.087 mmol) was added. 1,2-DCE (2 mL) was added. The reaction mixture was heated to 130 °C for 16 h. The reaction mixture was concentrated and purified by flash column chromatography (10:1 Hex:EtOAc) to give methyl 6-methyl-2-tosylisoindoline-4-carboxylate (**33**, 12.61 mg, 0.037 mmol, 42%) as a white crystalline solid.

^{1}H-NMR (500 MHz, $CDCl_3$): δ 7.78 (nfod, J_{app} = 8.0 Hz, 2H, *H2*), 7.72 (s, 1H, *H5′*), 7.30 (nfod, J_{app} = 7.9 Hz, 2H, *H3*), 7.16 (s, 1H, *H7′*), 4.87 (s, 2H, *H2′*), 4.60 (s, 2H, *H9′*), 3.89 (s, 3H, CO_2CH_3), 2.39 (s, 3H, *H5*), and 2.35 (s, 3H, *H6″*).

^{13}C-NMR{^{1}H} (126 MHz, $CDCl_3$): δ 166.4, 143.8, 138.3, 137.9, 135.7, 134.0, 130.2, 130.0, 127.73, 127.72, 125.2, 54.9, 53.2, 52.3, 21.6, and 21.2.

IR (neat): 2952 (C_{sp3}-H), 2923 (C_{sp3}-H), 2856 (C_{sp3}-H), and 1721(C=O) cm^{-1}.

HRMS (ESI-TOF): $[M+H]^+$ Calcd for $C_{18}H_{20}NO_4S^+$ 346.1108; Found 346.1094 (100%).

TLC: R_f 0.35 (5:1 Hex:EtOAc).

mp: 175–180 °C.

Methyl 6-Methyl-2-tosylisoindoline-4-carboxylate-7-*d* (33-d)

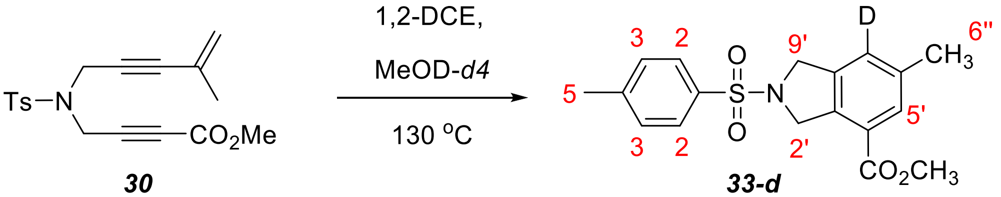

30 ***33-d***

In a 10 mL culture tube, equipped with a magnetic stir bar, **30** (21 mg, 0.064 mmol) was added. 1,2-DCE (1.8 mL) and CD_3OD (0.2 mL) were added. The reaction mixture was heated at 130 °C for 16 h. The reaction mixture was concentrated and purified by flash column chromatography (10:1 Hex:EtOAc) to give methyl 6-methyl-2-tosylisoindoline-4-carboxylate-7-*d* (**33-d**, 5.90 mg, 0.017 mmol, 27%) as a white crystalline solid.

^{1}H-NMR (500 MHz, $CDCl_3$): δ 7.78 (nfod, J_{app} = 8.3 Hz, 2H, *H2*), 7.72 (q, J = 0.8 Hz, 1H, *H5'*), 7.30 (nfod, J_{app} = 8.2 Hz, 2H, *H3*), 7.16 (s, 0.1H, H7'), 4.87 (nfot, J_{app} = 2.1 Hz, 2H, *H2'*), 4.60 (nfot, J_{app} = 2.2 Hz, 2H, *H9'*), 3.89 (s, 3H, CO_2CH_3), 2.39 (s, 3H, C4CH_3), and 2.35 (d, J = 0.9 Hz, 3H, C6'CH_3).

^{13}C-NMR{^{1}H} (126 MHz, $CDCl_3$): δ 166.4, 143.8, 138.2, 137.8, 135.7, 134.0, 130.2, 130.0, 127.74, 125.3, 54.9, 53.2, 52.3, 21.7, and 21.1 (no resonance observed at 127.72, the largely deuterated C7')

IR (neat): 2952 (C_{sp3}-H), 2921 (C_{sp3}-H), 2850 (C_{sp3}-H), and 1721(C=O) cm^{-1}.

HRMS (ESI-TOF): $[M+H]^+$ Calcd for $C_{18}H_{19}DNO_4S^+$ 347.1170; Found 347.1155 (100%).

TLC: R_f 0.35 (5:1 Hex:EtOAc).

mp: 186–190 °C.

III. DP4+ NMR analysis of structures 17 (isomer 1) and S5 (isomer 2)

The two possible structures for the minor isomer were **17** and **S5** (Figure S1). These were subjected to a DP4+[3] analysis to compare with the sets or proton and carbon chemical shift experimental data. Each of structure was first subjected to a Monte Carlo multi conformational search in MacroModel (using Maestro) with the OPLS_2005 molecular mechanics (MM) force field within the Schrodinger software suite (Release 2020-4).[4] Both conformational search outputs resulted in only a few conformations for each isomer. These were used as the geometries in the .gjf input files for DFT optimization in Gaussian 16.[5] The M062X functional and 6-31+G(d,p) basis set with PCM (chloroform) solvation were used. The literature protocol describing this process as well as the Python scripts used to streamline the workflow were followed.[6] The structure (CYLview image) and computed energies of each optimized structure (one for isomer 1 and two for isomer 2) are given below. The Gaussian .out files for each of these three structure have been uploaded as a separate .zip file. The .out file of any optimized structures can be opened directly in a structure visualization program [e.g., GaussView, CYLview (https://www.cylview.org), or Avogadro 2 (https://www.openchemistry.org/projects/avogadro2)], which will automatically and conveniently extract and open an interrogatable 3D model of the final optimized structure.

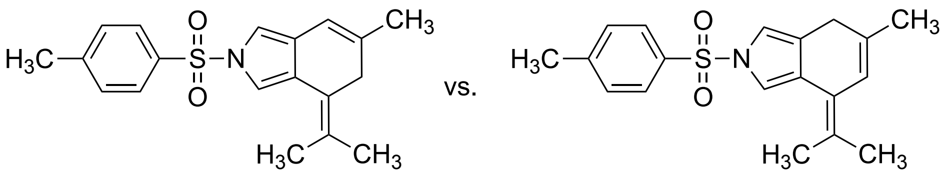

17
(isomer 1)

S5
(isomer 2)

Figure S1. Possible structures considered for the minor product.

"The NMR shielding tensors of all of the protons and carbons in each of the resulting unique conformers were calculated using the Sarotti recommended functional (mPW1PW91) and basis set [6-31+G(d,p)] with chloroform solvation modeling (IEFPCM). Boltzmann weighting was then used to compute the averaged chemical shift for each proton and carbon. These were used in the final DP4+ (spreadsheet) analysis; the following website provides clear instructions for how to proceed with that analysis: https://sarotti-nmr.weebly.com [accessed March 10, 2025]. The results are summarized below"[7] in the screenshot of the DP4+ Excel spreadsheet. The analysis clearly indicates that structure **17** is much more consistent than **S5** with the data of the side product.

Functional		Solvent?		Basis Set		Type of Data	
mPW1PW91		PCM		6-31+G(d,p)		Shielding Tensors	
		DP4+	100.00%	0.00%	–	–	–
Nuclei	sp2?	Experimenta	Isomer 1	Isomer 2	Isomer 3	Isomer 4	Isomer 5
H	x	6.82	24.6	24.4			
H	x	7.03	24.3	24.2			
H	x	6.07	25.2	24.9			
H		3.01	28.5	28.3			
H		1.81	29.8	29.8			
H		1.82	29.8	29.6			
H		1.93	29.6	29.6			
H	x	7.72	23.4	23.4			
H	x	7.25	23.9	23.9			
H		2.38	29.2	29.2			
C	x	126.78	75.2	74.2			
C	x	124	70.51	72.24			
C	x	113.3	86.72	84.36			
C	x	117.8	81.39	82.82			
C	x	120.6	74.95	75.60			
C	x	114	84.34	77.07			
C	x	136.6	58.09	64.24			
C		35.7	158.25	165.28			
C		23.4	171.77	171.07			
C	x	129.4	64.90	65.22			
C		22.3	174.00	174.12			
C		24.1	171.88	171.10			
C	x	136.3	60.51	60.41			
C	x	126.84	71.94	71.91			
C	x	130	69.77	69.80			
C	x	144.7	51.7051	51.66091137			
C		21.7	173.5454	173.785217			

Figure S2. Screenshot of DP4+ NMR analysis (chloroform for the PCM solvation).

Geometry and energies [in Hartree (au)] for 17 (isomer1)-conf-1

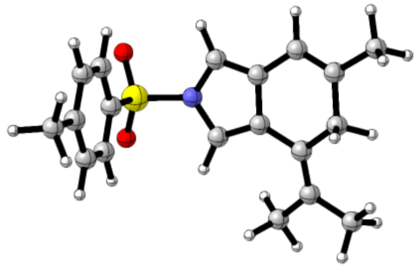

Sum of electronic and zero-point Energies= -1339.228119
Sum of electronic and thermal Energies= -1339.205652
Sum of electronic and thermal Enthalpies= -1339.204708
Sum of electronic and thermal Free Energies= -1339.282376

Geometry and energies [in Hartree (au)] for S5 (isomer2)-conf-1

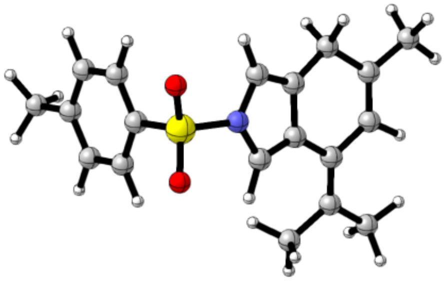

Sum of electronic and zero-point Energies= -1339.229191
Sum of electronic and thermal Energies= -1339.206768
Sum of electronic and thermal Enthalpies= -1339.205824
Sum of electronic and thermal Free Energies= -1339.283971

Geometry and energies [in Hartree (au)] for S5 (isomer2)-conf-2

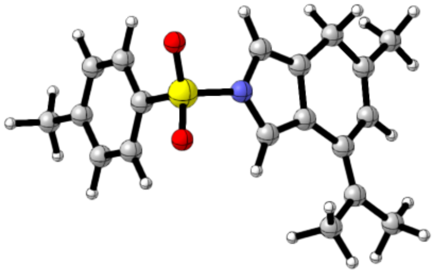

Sum of electronic and zero-point Energies= -1339.228867
Sum of electronic and thermal Energies= -1339.206431
Sum of electronic and thermal Enthalpies= -1339.205487
Sum of electronic and thermal Free Energies= -1339.284073

IV. Supporting Information References

1 Hoye, T. R.; Hanson, P. R; Vyvyan, J. R. A Practical guide to first-order multiplet analysis In ^{1}H NMR spectroscopy. *J. Org. Chem.* **1994**, *59*, 4096–4103. [https://pubs.acs.org/doi/abs/10.1021/jo00094a018]

2 Hoye, T. R.; Zhao, H. A method for easily determining coupling constant (*J*) values: An addendum to "A practical guide to first-order multiplet analysis in ^{1}H NMR spectroscopy." *J. Org. Chem.* **2002**, *67*, 4014–4016. [https://pubs.acs.org/doi/10.1021/jo001139v]

3 Grimblat, N.; Zanardi, M. M.; Sarotti, A. M. Beyond DP4: An improved probability for the stereochemical assignment of isomeric compounds using quantum chemical calculations of NMR shifts. *J. Org. Chem.* **2015**, *80*, 12526–12534. [https://pubs.acs.org/doi/10.1021/acs.joc.5b02396]

4 Schrödinger Release 2020-4, Maestro, Schrödinger, LLC, New York, NY, 2020.

5 M. J. Frisch, G. W. Trucks, H. B. Schlegel, G. E. Scuseria, M. A. Robb, J. R. Cheeseman, G. Scalmani, V. Barone, G. A. Petersson, H. Nakatsuji, X. Li, M. Caricato, A. V. Marenich, J. Bloino, B. G. Janesko, R. Gomperts, B. Mennucci, H. P. Hratchian, J. V. Ortiz, A. F. Izmaylov, J. L. Sonnenberg, D. Williams-Young, F. Ding, F. Lipparini, F. Egidi, J. Goings, B. Peng, A. Petrone, T. Henderson, D. Ranasinghe, V. G. Zakrzewski, J. Gao, N. Rega, G. Zheng, W. Liang, M. Hada, M. Ehara, K. Toyota, R. Fukuda, J. Hasegawa, M. Ishida, T. Nakajima, Y. Honda, O. Kitao, H. Nakai, T. Vreven, K. Throssell, J. A. Montgomery, Jr., J. E. Peralta, F. Ogliaro, M. J. Bearpark, J. J. Heyd, E. N. Brothers, K. N. Kudin, V. N. Staroverov, T. A. Keith, R. Kobayashi, J. Normand, K. Raghavachari, A. P. Rendell, J. C. Burant, S. S. Iyengar, J. Tomasi, M. Cossi, J. M. Millam, M. Klene, C. Adamo, R. Cammi, J. W. Ochterski, R. L. Martin, K. Morokuma, O. Farkas, J. B. Foresman and D. J. Fox, Gaussian 16, Revision C.01, Gaussian, Inc., Wallingford CT, 2016.

6 (a) Willoughby, P. H.; Jansma, M. J.; Hoye, T. R. A guide to small-molecule structure assignment through computation of (^{1}H and ^{13}C) NMR chemical shifts, *Nat. Protoc*. **2014**, *9*, 643–660. [https://www.nature.com/articles/nprot.2014.042] (b) See the following Addendum for more robust versions of the Python scripts: Willoughby, P. H.; Jansma, M. J.; Hoye, T. R. Addendum: A guide to small-molecule structure assignment through computation of (^{1}H and ^{13}C) NMR chemical shifts, *Nat. Protoc.* **2020**, *15*, 2277–2277. [https://www.nature.com/articles/s41596-020-0293-9]

7 Kevorkian, P. V.; Sneddon, D. S.; Ritts, C. B.; Hoye, T. R. Phosphorane-promoted C−C coupling during aryne annulations. *Angew. Chem. Int. Ed.* **2024**, *63*, e202318774. [https://onlinelibrary.wiley.com/doi/10.1002/anie.202318774]

V. Copies of NMR Spectra

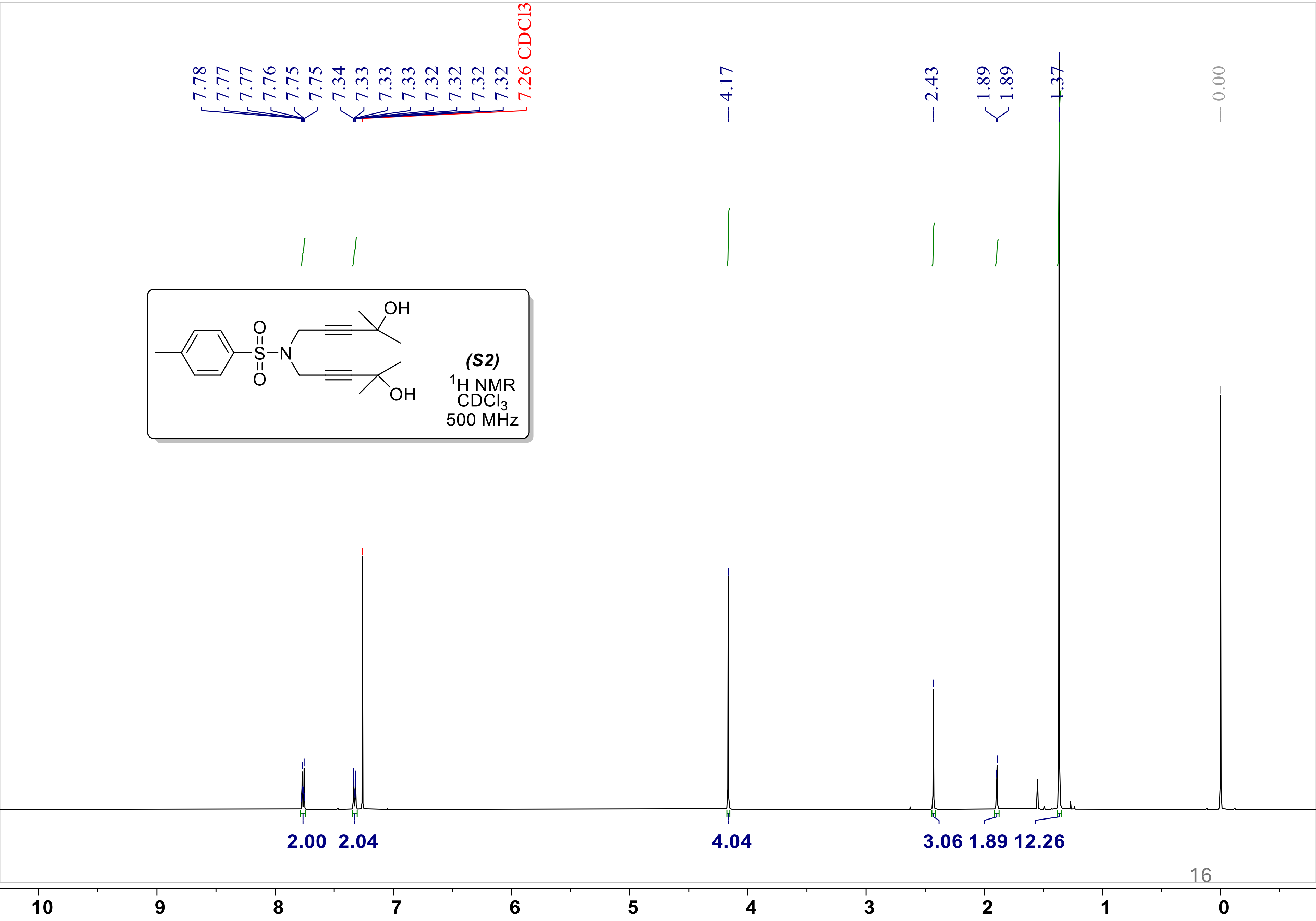

7.78
7.77
7.77
7.76
7.75
7.75
7.34
7.33
7.33
7.33
7.32
7.32
7.32
7.32
7.26 CDCl3
4.17
2.43
1.89
1.89
1.37
0.00
OH
O
S
N
O
OH
(S2)
1H NMR
CDCl3
500 MHz
2.00
2.04
4.04
3.06
1.89
12.26
10
9
8
7
6
5
4
3
2
1
0

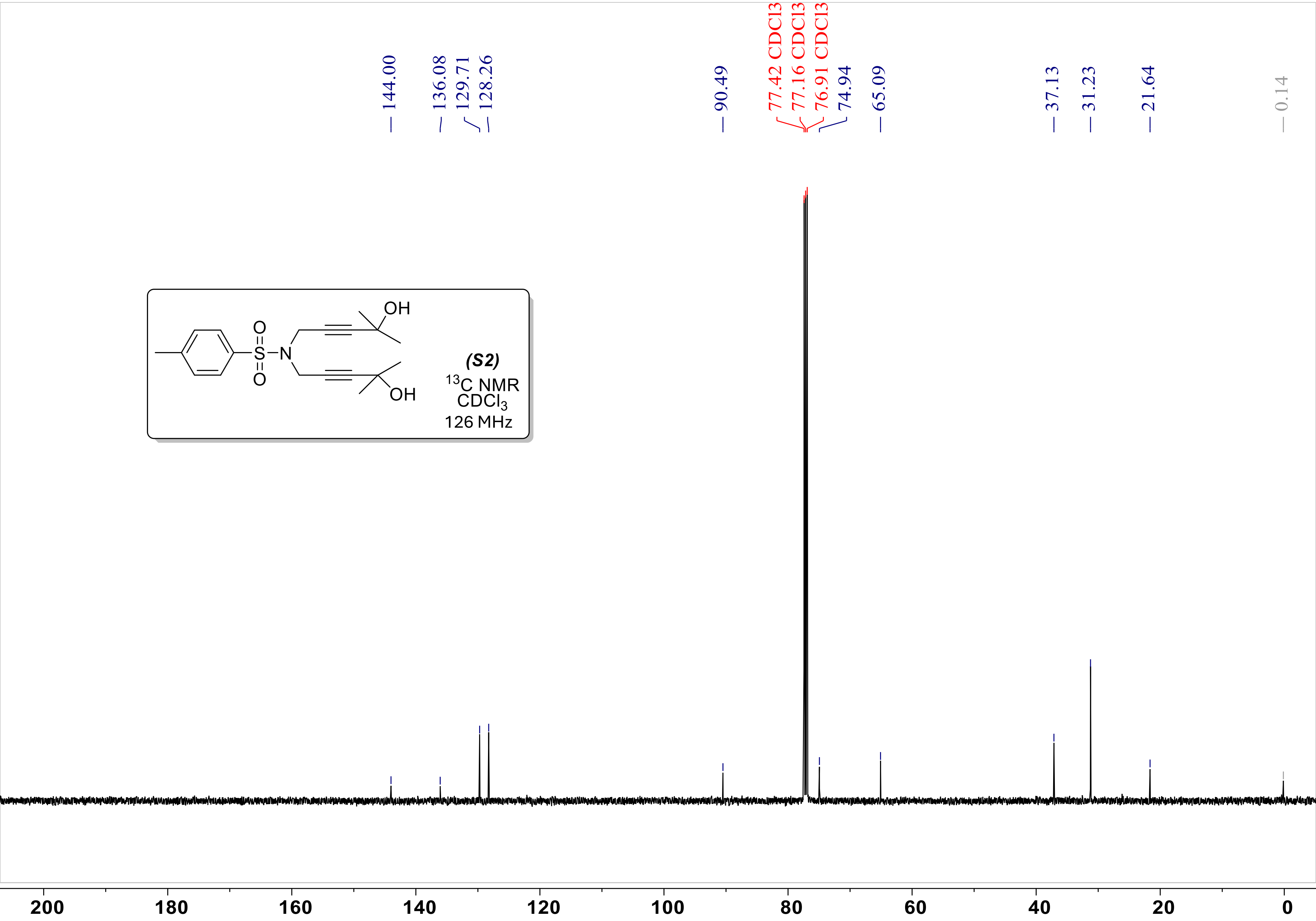
144.00
136.08
129.71
128.26
90.49
77.42 CDCl3
77.16 CDCl3
76.91 CDCl3
74.94
65.09
37.13
31.23
21.64
0.14
OH
O
S
N
O
OH
(S2)
13C NMR
CDCl3
126 MHz
200
180
160
140
120
100
80
60
40
20
0

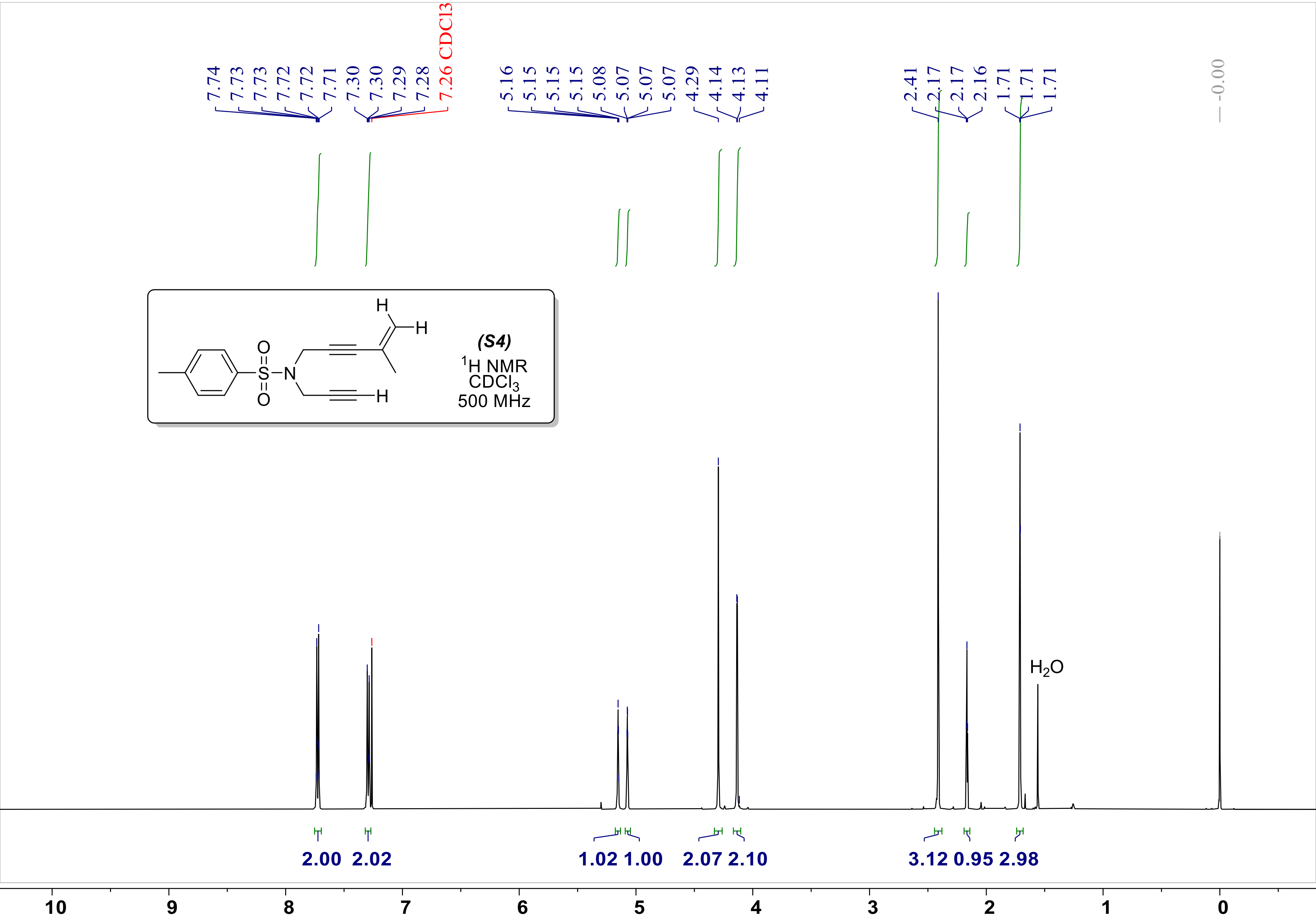
7.74 7.73 7.73 7.72 7.72 7.71 7.30 7.30 7.29 7.28 7.26 CDCl3
5.16 5.15 5.15 5.15 5.08 5.07 5.07 5.07 4.29 4.14 4.13 4.11
2.41 2.17 2.17 2.16 1.71 1.71 1.71
-0.00
(S4)
1H NMR
CDCl3
500 MHz
H2O
2.00 2.02 1.02 1.00 2.07 2.10 3.12 0.95 2.98
10 9 8 7 6 5 4 3 2 1 0

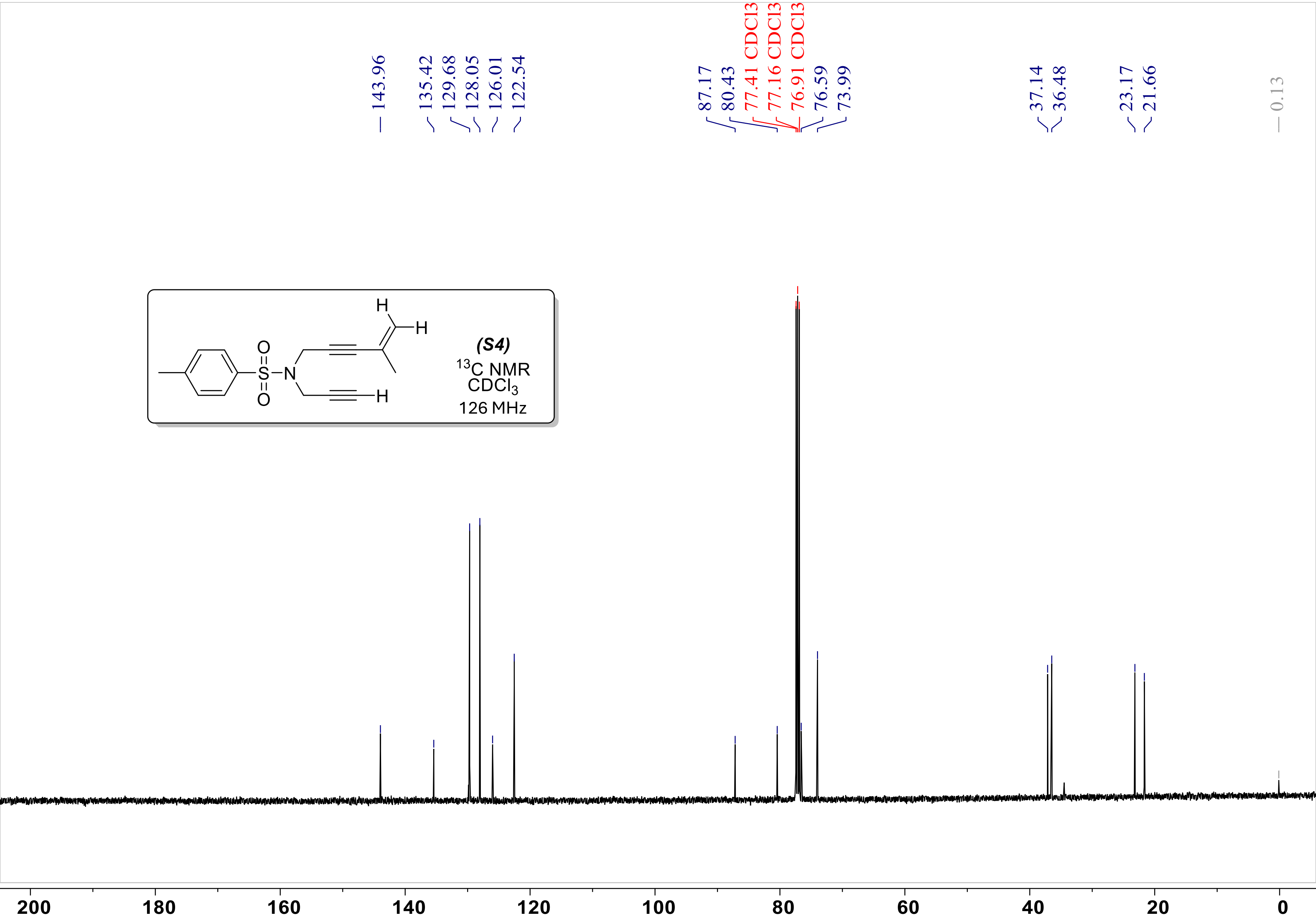
143.96
135.42
129.68
128.05
126.01
122.54
87.17
80.43
77.41 CDCl3
77.16 CDCl3
76.91 CDCl3
76.59
73.99
37.14
36.48
23.17
21.66
0.13
(S4)
13C NMR
CDCl3
126 MHz
200
180
160
140
120
100
80
60
40
20
0

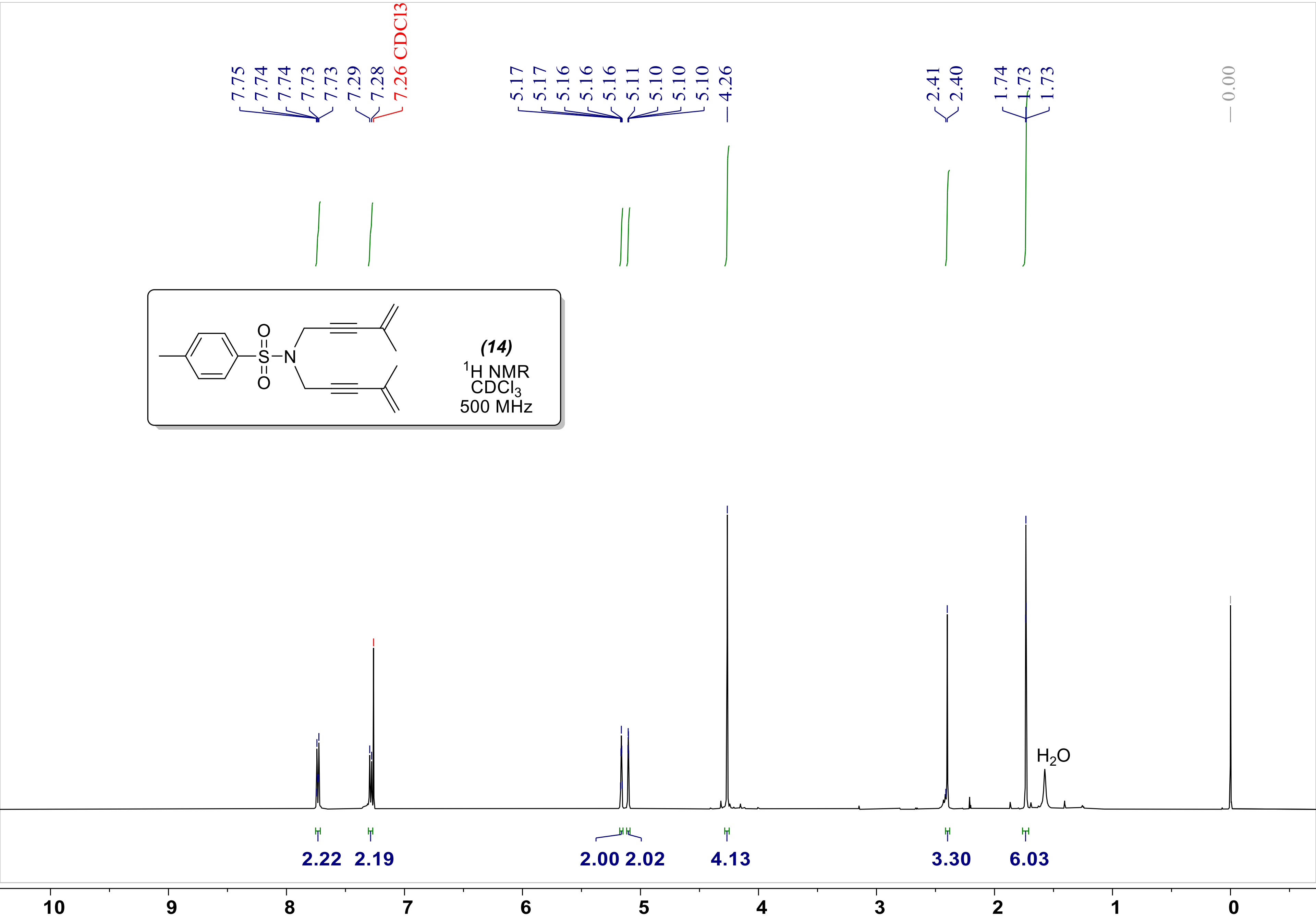
7.75 7.74 7.74 7.73 7.73 7.29 7.28 7.26 CDCl3
5.17 5.17 5.16 5.16 5.16 5.11 5.10 5.10 5.10 4.26
2.41 2.40 1.74 1.73 1.73 0.00
(14)
^1H NMR
$CDCl_3$
500 MHz
H_2O
2.22 2.19 2.00 2.02 4.13 3.30 6.03
10 9 8 7 6 5 4 3 2 1 0

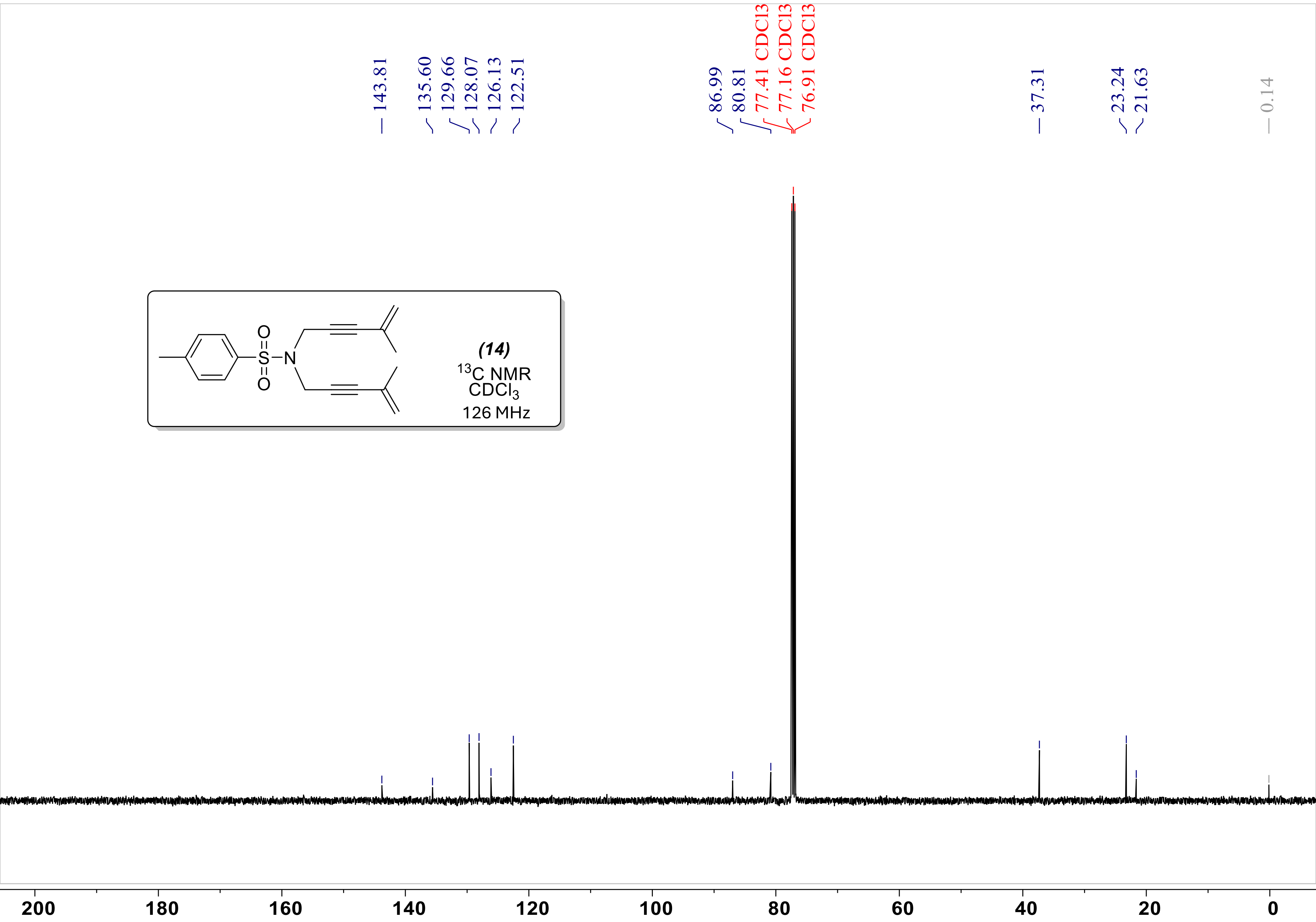
143.81
135.60
129.66
128.07
126.13
122.51
86.99
80.81
77.41 CDCl3
77.16 CDCl3
76.91 CDCl3
37.31
23.24
21.63
0.14
(14)
13C NMR
CDCl3
126 MHz
200
180
160
140
120
100
80
60
40
20
0

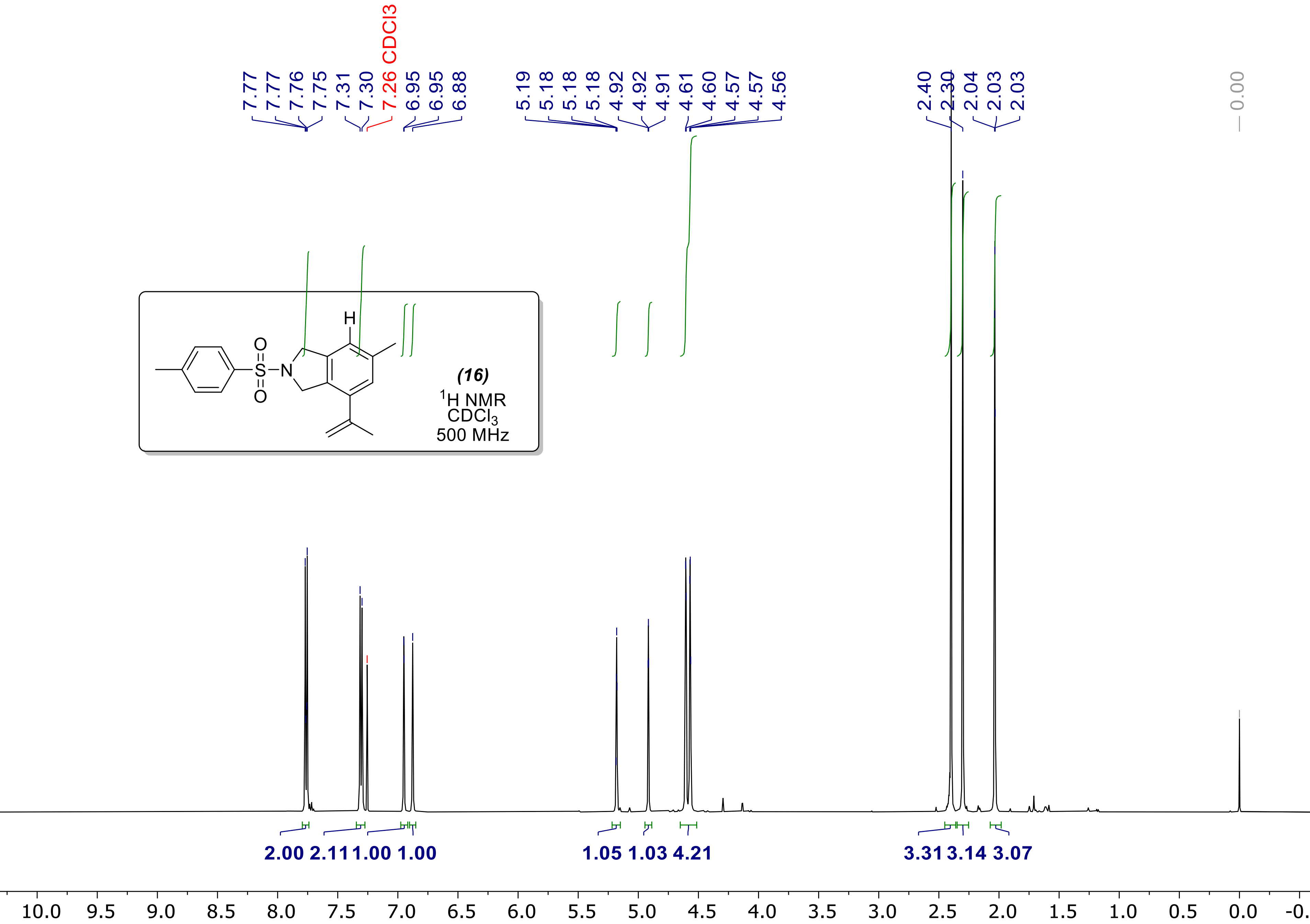
7.77 7.77 7.76 7.75 7.31 7.30 7.26 CDCl3 6.95 6.95 6.88
5.19 5.18 5.18 5.18 4.92 4.92 4.91 4.61 4.60 4.57 4.57 4.56
2.40 2.30 2.04 2.03 2.03
0.00
H
O
S
N
O
(16)
1H NMR
CDCl3
500 MHz
2.00 2.11 1.00 1.00
1.05 1.03 4.21
3.31 3.14 3.07
10.0 9.5 9.0 8.5 8.0 7.5 7.0 6.5 6.0 5.5 5.0 4.5 4.0 3.5 3.0 2.5 2.0 1.5 1.0 0.5 0.0

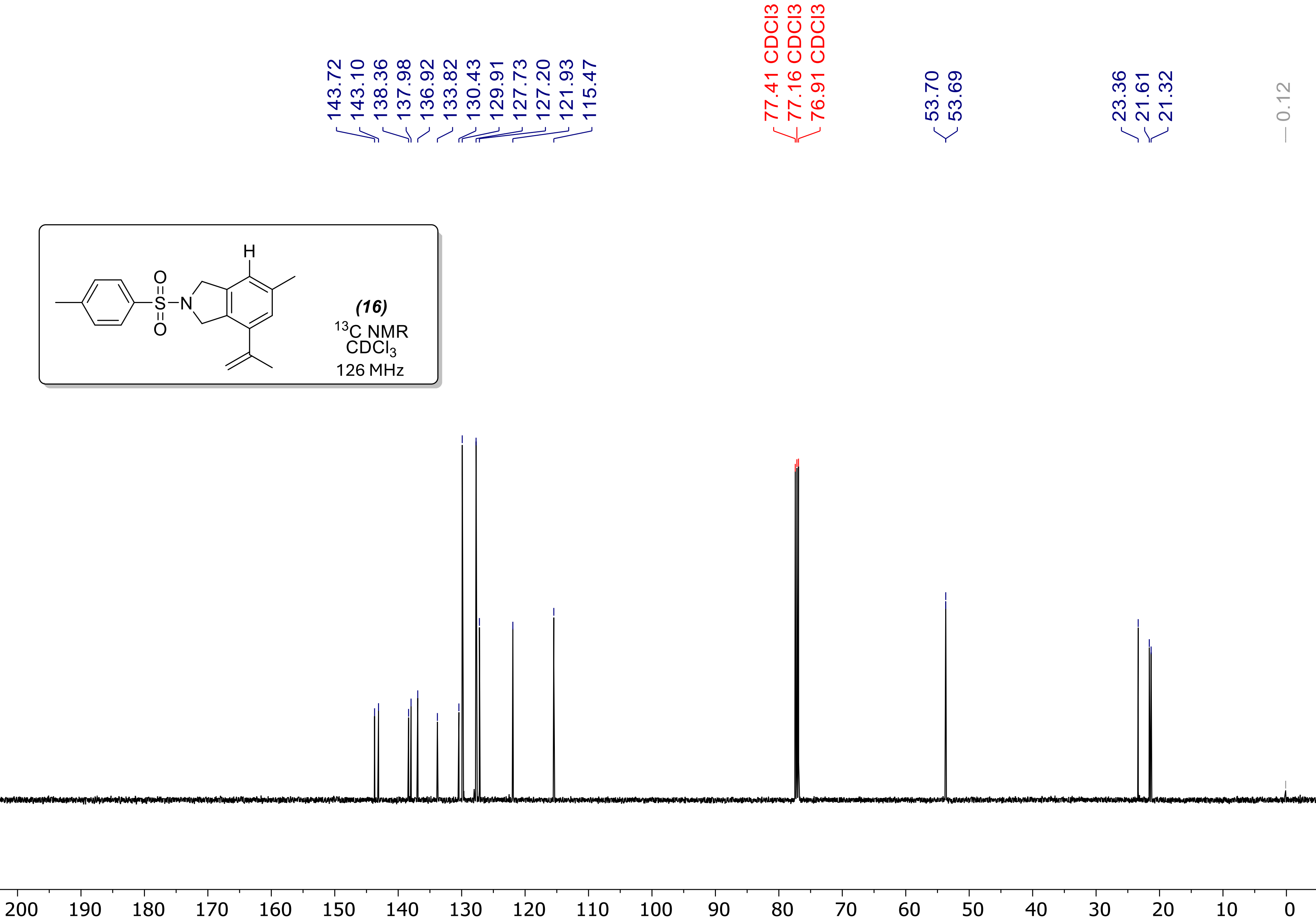
143.72
143.10
138.36
137.98
136.92
133.82
130.43
129.91
127.73
127.20
121.93
115.47
77.41 CDCl3
77.16 CDCl3
76.91 CDCl3
53.70
53.69
23.36
21.61
21.32
0.12
H
O
S
N
O
(16)
13C NMR
CDCl3
126 MHz
200 190 180 170 160 150 140 130 120 110 100 90 80 70 60 50 40 30 20 10 0

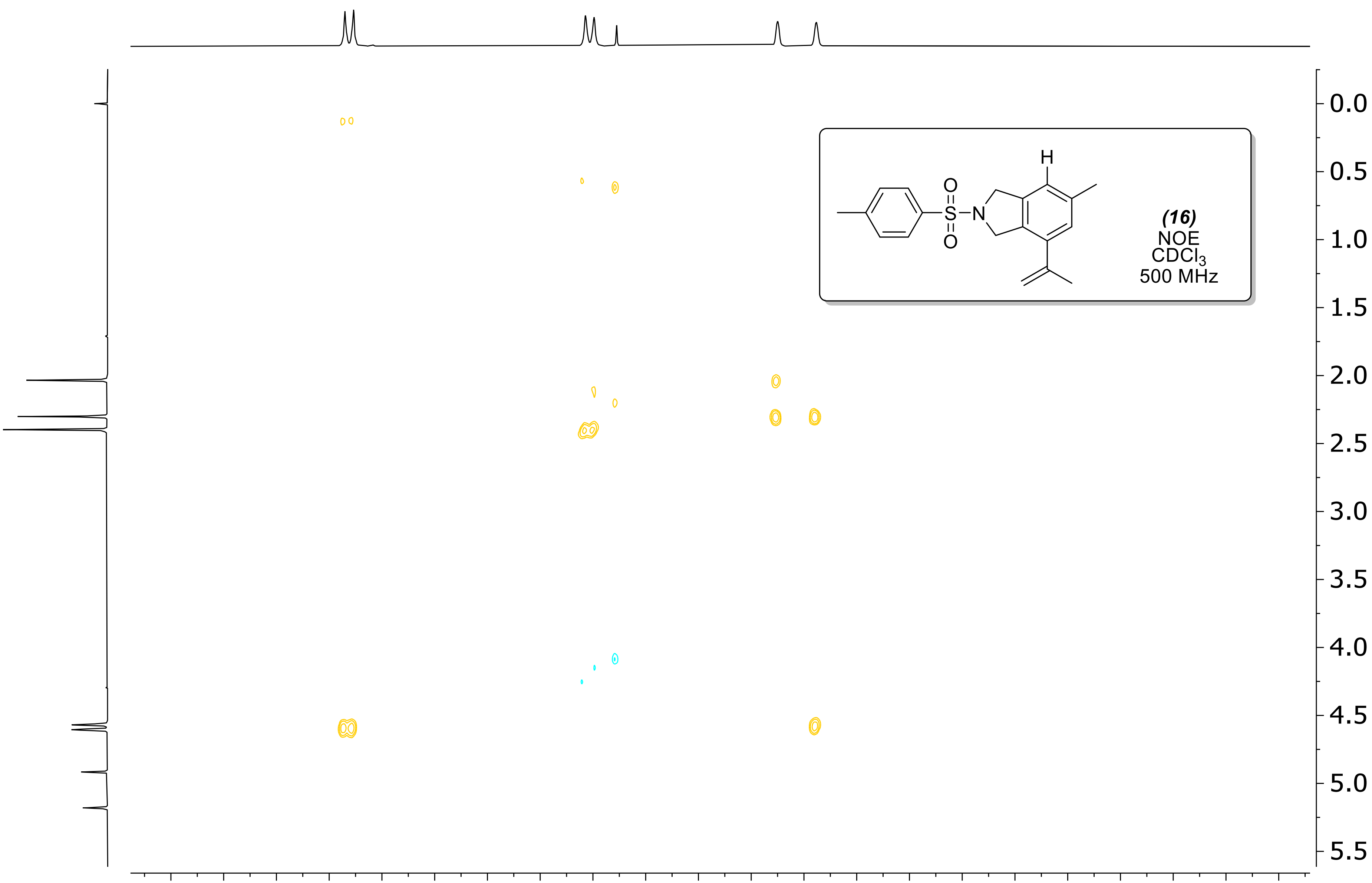
(16)
NOE
$CDCl_3$
500 MHz
O
S
N
H
8.1 8.0 7.9 7.8 7.7 7.6 7.5 7.4 7.3 7.2 7.1 7.0 6.9 6.8 6.7 6.6 6.5 6.4 6.3 6.2 6.1 6.0
0.0 0.5 1.0 1.5 2.0 2.5 3.0 3.5 4.0 4.5 5.0 5.5

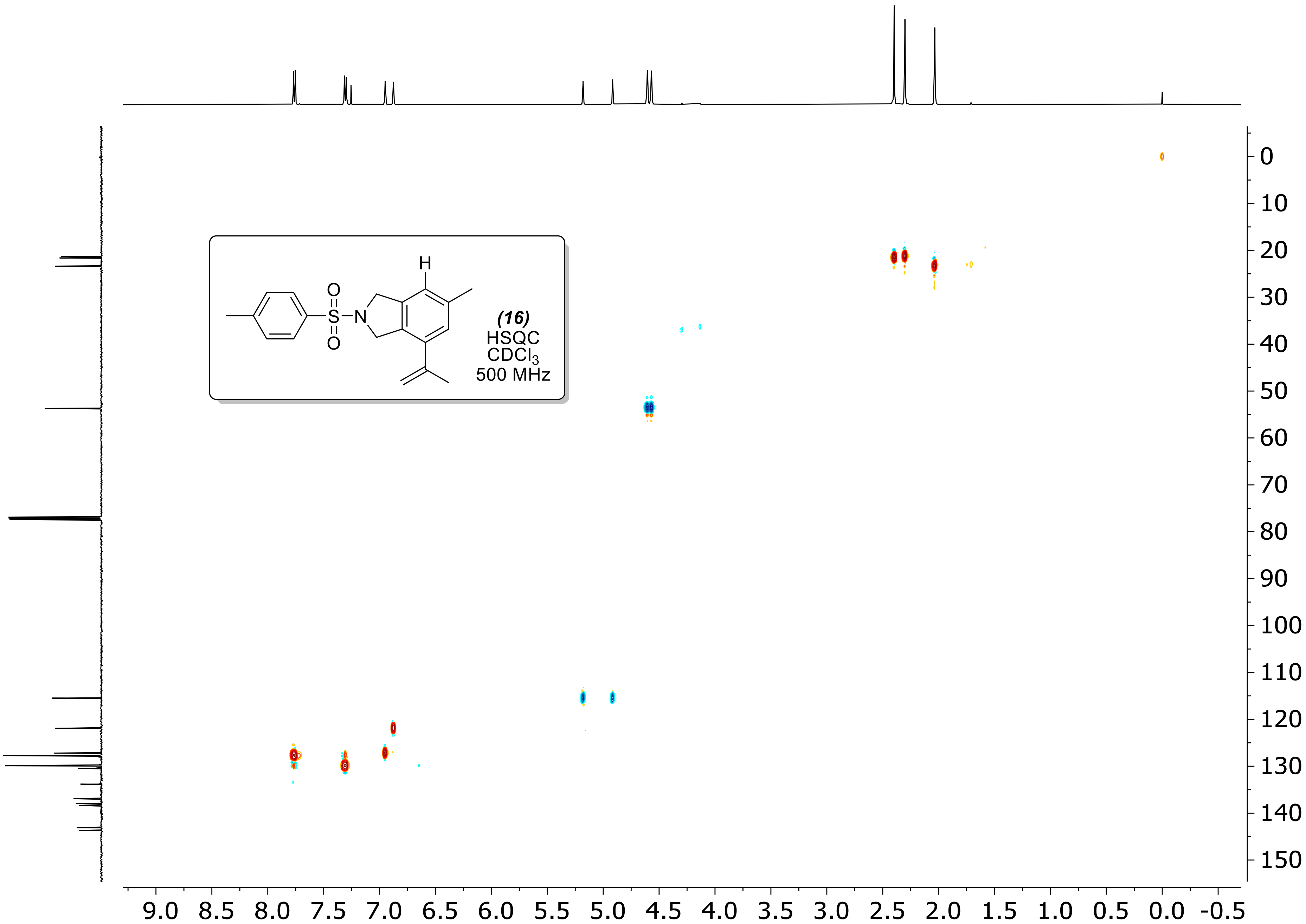
(16)
HSQC
$CDCl_3$
500 MHz
0
10
20
30
40
50
60
70
80
90
100
110
120
130
140
150
9.0 8.5 8.0 7.5 7.0 6.5 6.0 5.5 5.0 4.5 4.0 3.5 3.0 2.5 2.0 1.5 1.0 0.5 0.0 -0.5

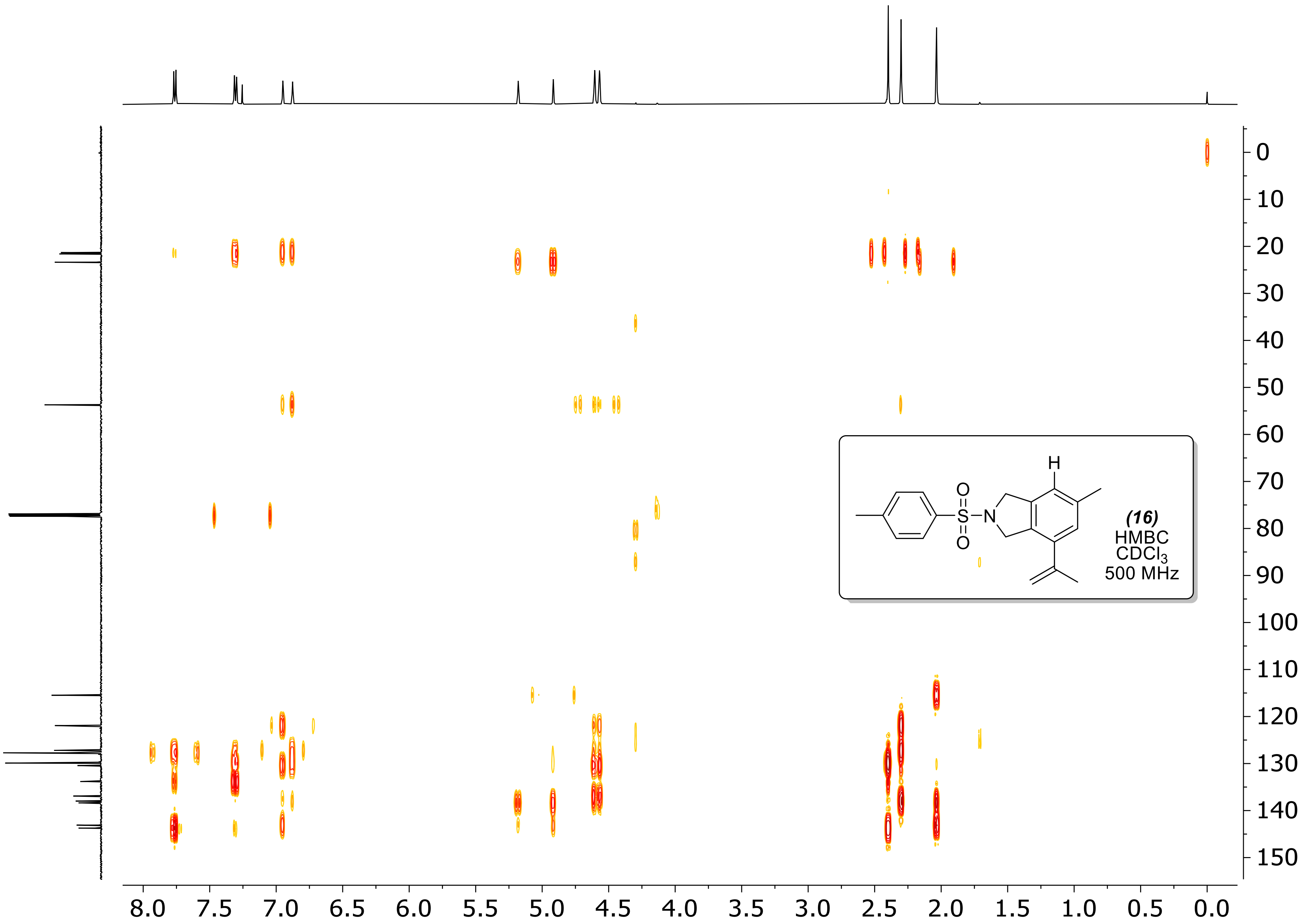
(16)
HMBC
CDCl3
500 MHz
8.0 7.5 7.0 6.5 6.0 5.5 5.0 4.5 4.0 3.5 3.0 2.5 2.0 1.5 1.0 0.5 0.0
0 10 20 30 40 50 60 70 80 90 100 110 120 130 140 150

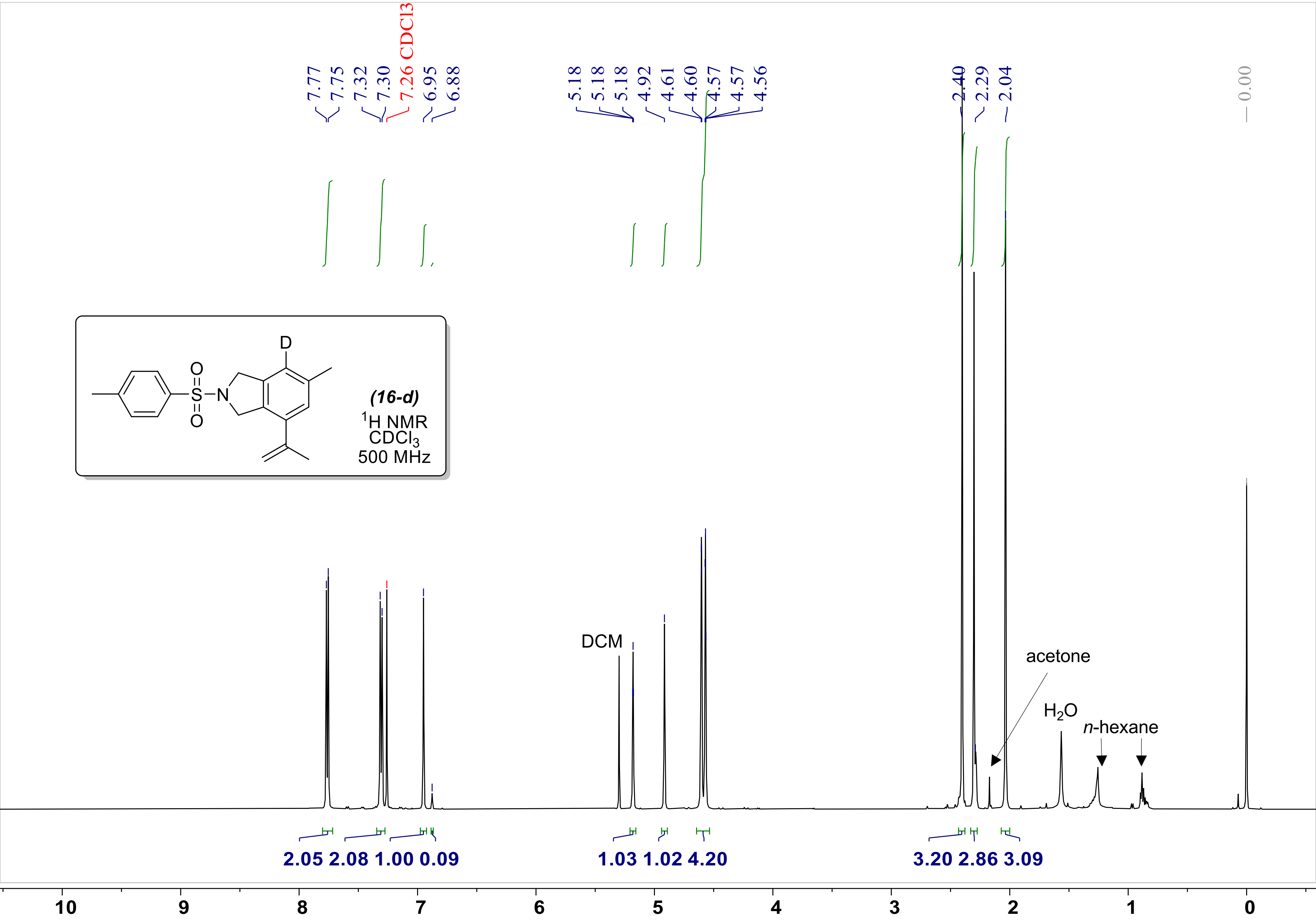
7.77
7.75
7.32
7.30
7.26 CDCl3
6.95
6.88
5.18
5.18
5.18
4.92
4.61
4.60
4.57
4.57
4.56
2.40
2.29
2.04
0.00
D
O
S
N
O
(16-d)
1H NMR
CDCl3
500 MHz
DCM
acetone
H2O
n-hexane
2.05 2.08 1.00 0.09
1.03 1.02 4.20
3.20 2.86 3.09
10
9
8
7
6
5
4
3
2
1
0

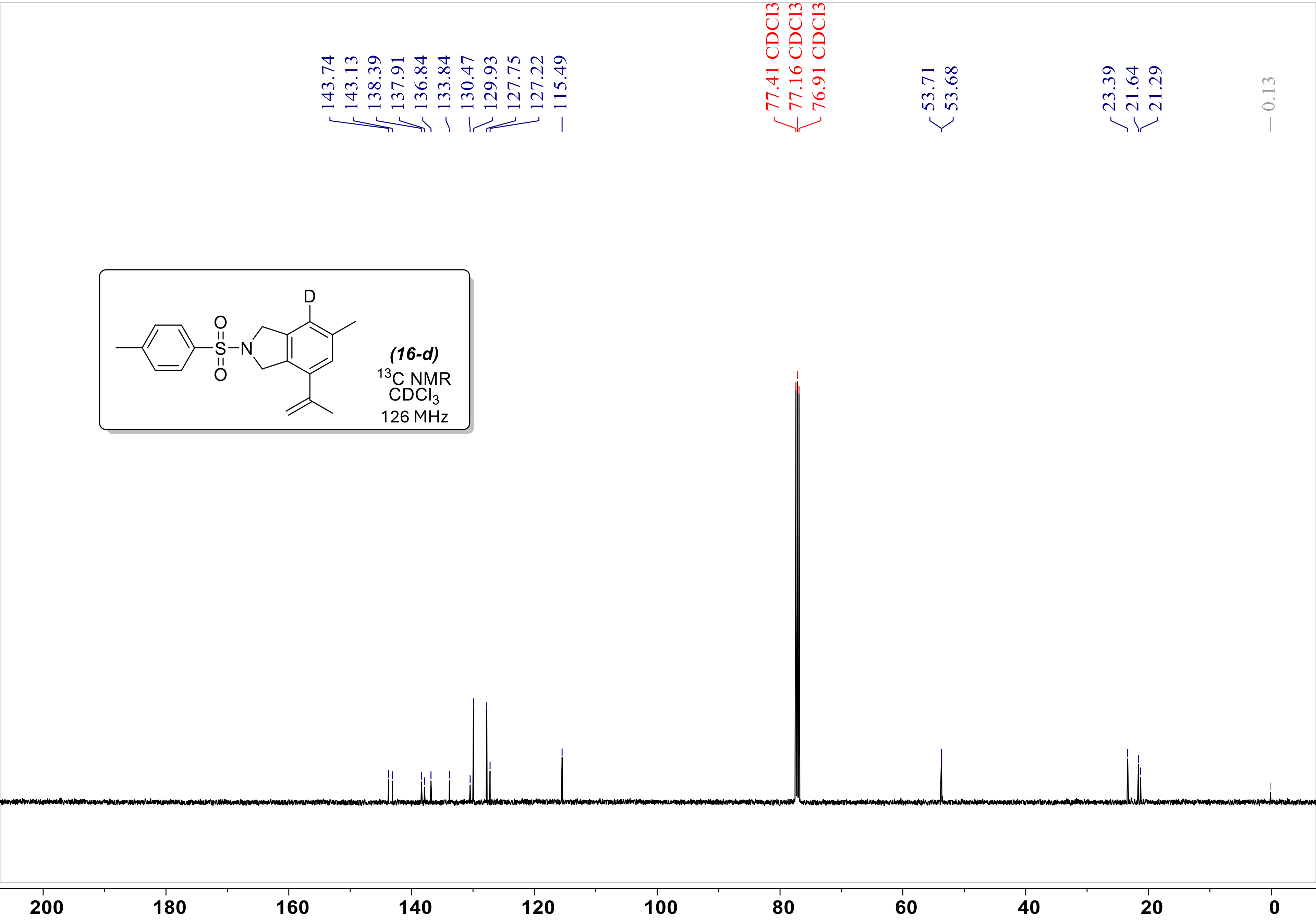
143.74
143.13
138.39
137.91
136.84
133.84
130.47
129.93
127.75
127.22
115.49
77.41 CDCl3
77.16 CDCl3
76.91 CDCl3
53.71
53.68
23.39
21.64
21.29
0.13
D
O
S
N
O
(16-d)
13C NMR
CDCl3
126 MHz
200
180
160
140
120
100
80
60
40
20
0

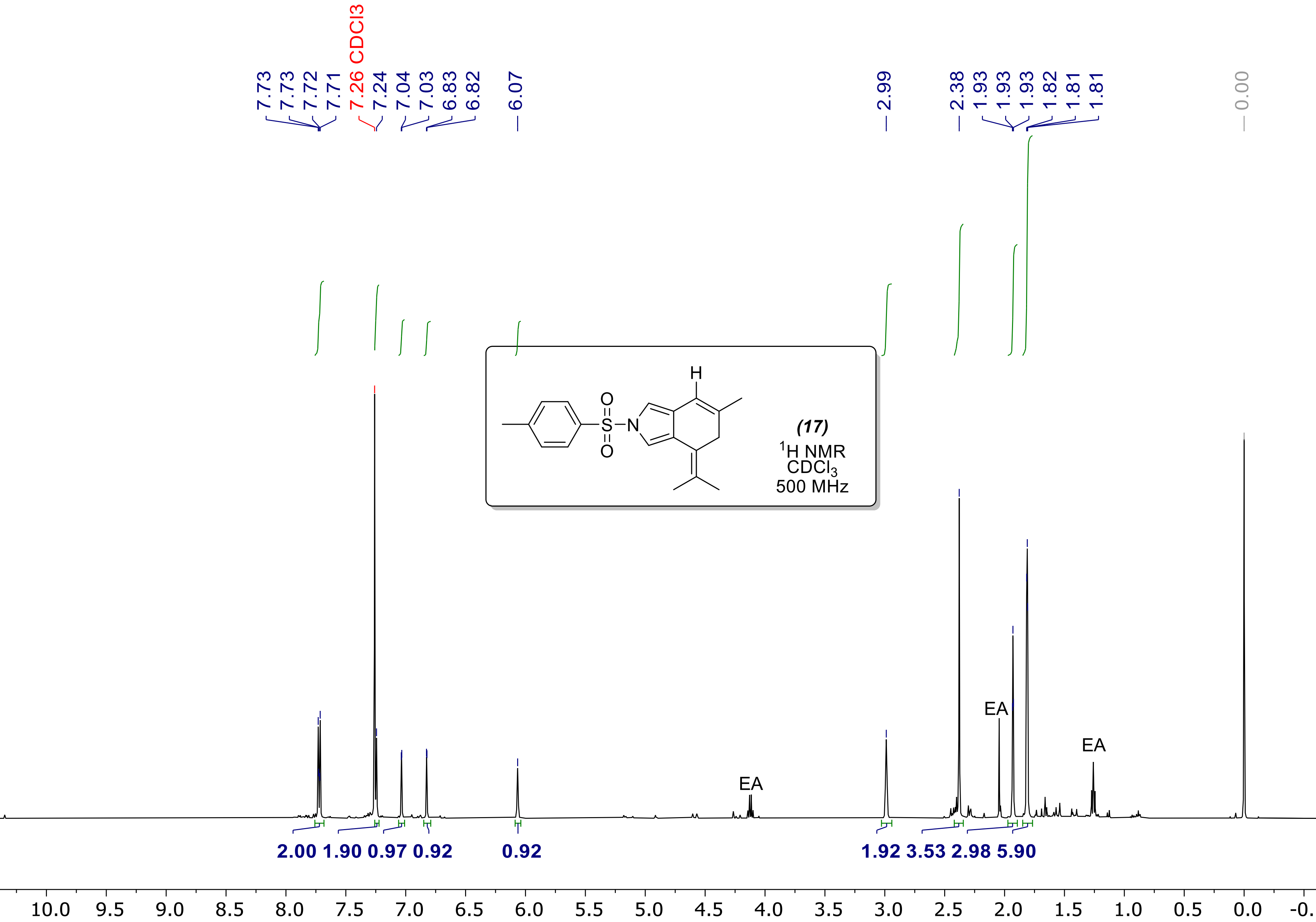
7.73
7.73
7.72
7.71
7.26 CDCl3
7.24
7.04
7.03
6.83
6.82
6.07
2.99
2.38
1.93
1.93
1.93
1.82
1.81
1.81
0.00
(17)
^{1}H NMR
$CDCl_3$
500 MHz
EA
EA
EA
2.00 1.90 0.97 0.92
0.92
1.92 3.53 2.98 5.90
10.0 9.5 9.0 8.5 8.0 7.5 7.0 6.5 6.0 5.5 5.0 4.5 4.0 3.5 3.0 2.5 2.0 1.5 1.0 0.5 0.0

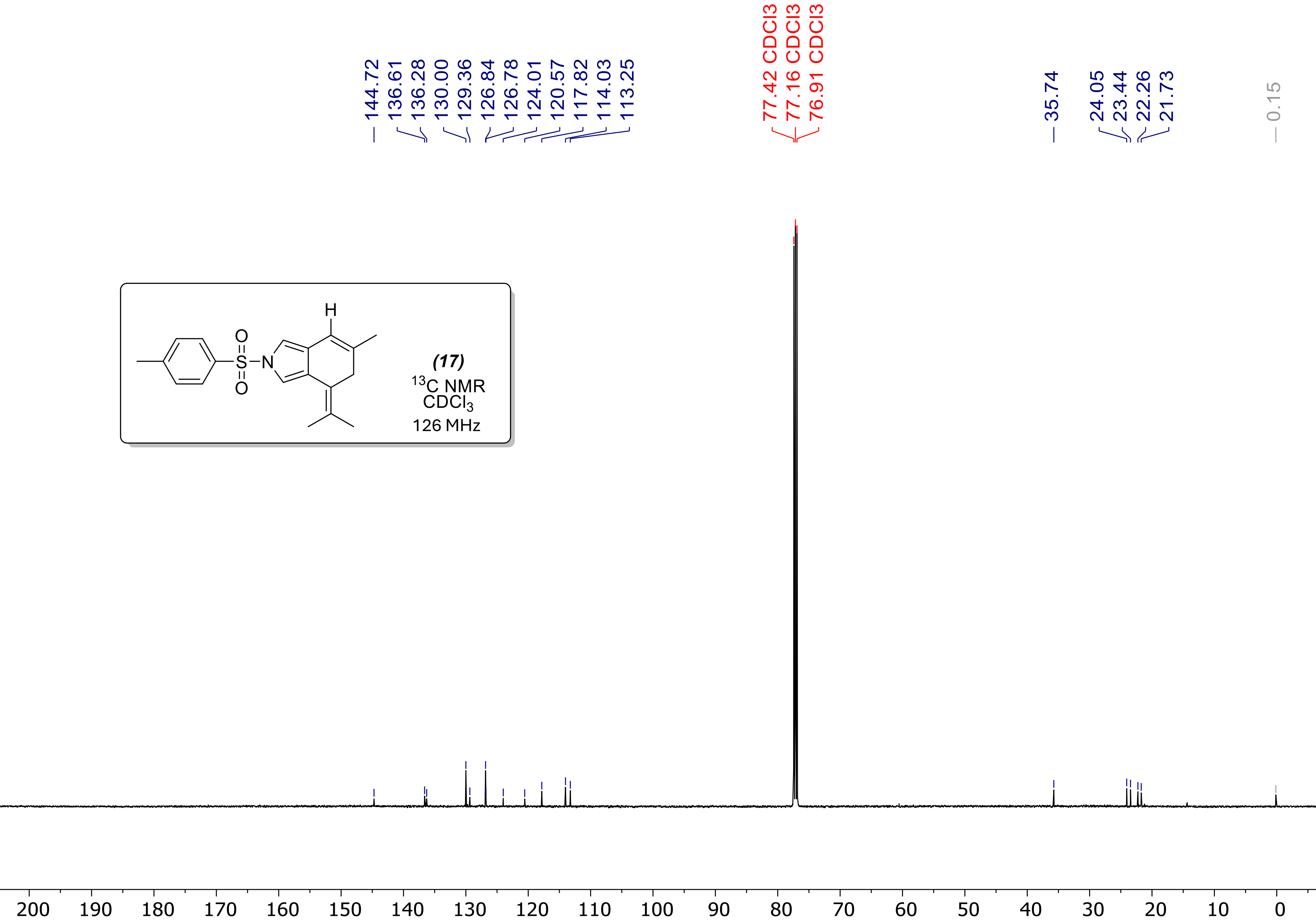
144.72
136.61
136.28
130.00
129.36
126.84
126.78
124.01
120.57
117.82
114.03
113.25
77.42 CDCl3
77.16 CDCl3
76.91 CDCl3
35.74
24.05
23.44
22.26
21.73
0.15
(17)
13C NMR
CDCl3
126 MHz
200 190 180 170 160 150 140 130 120 110 100 90 80 70 60 50 40 30 20 10 0

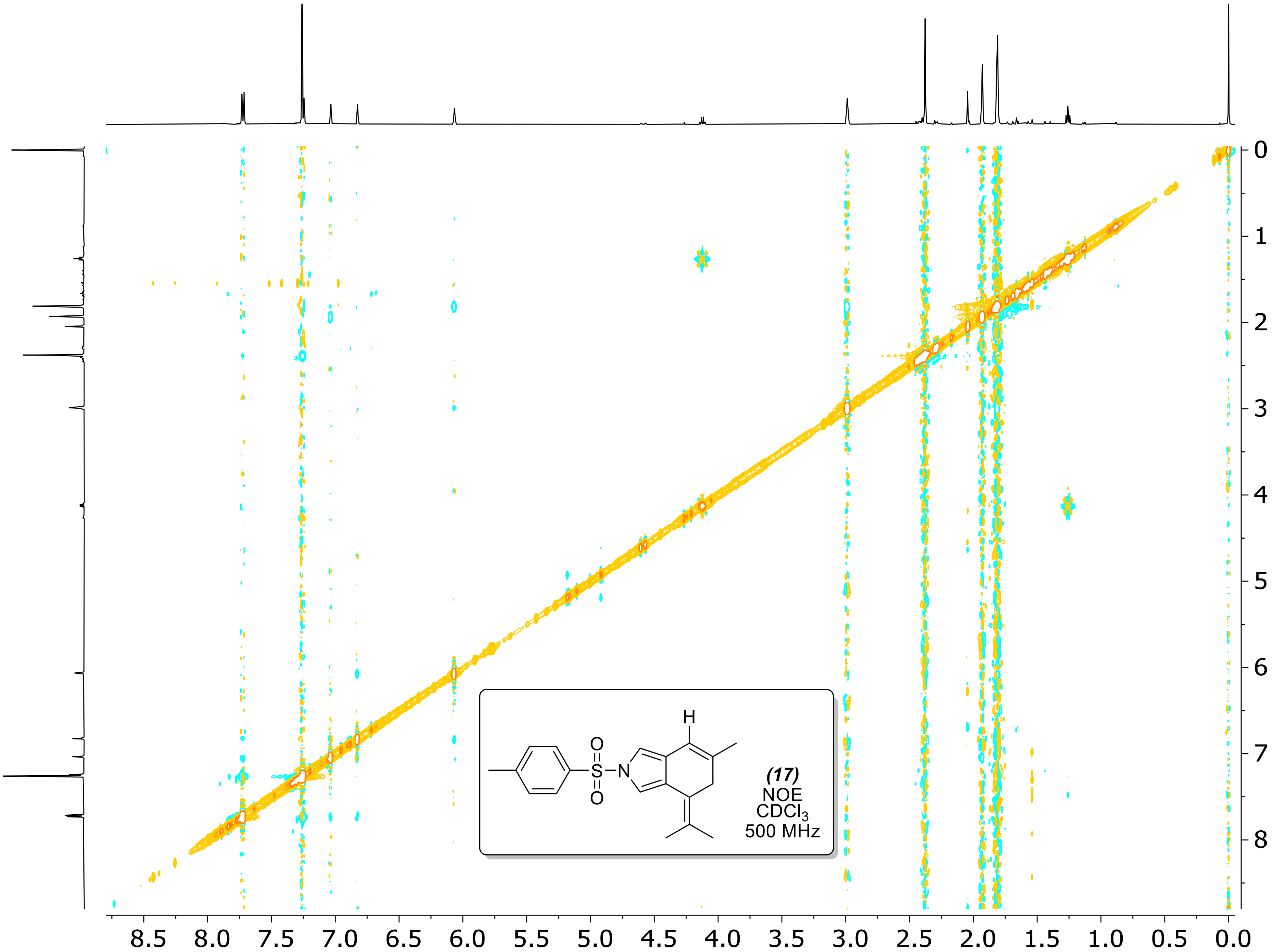

(17)
NOE
$CDCl_3$
500 MHz

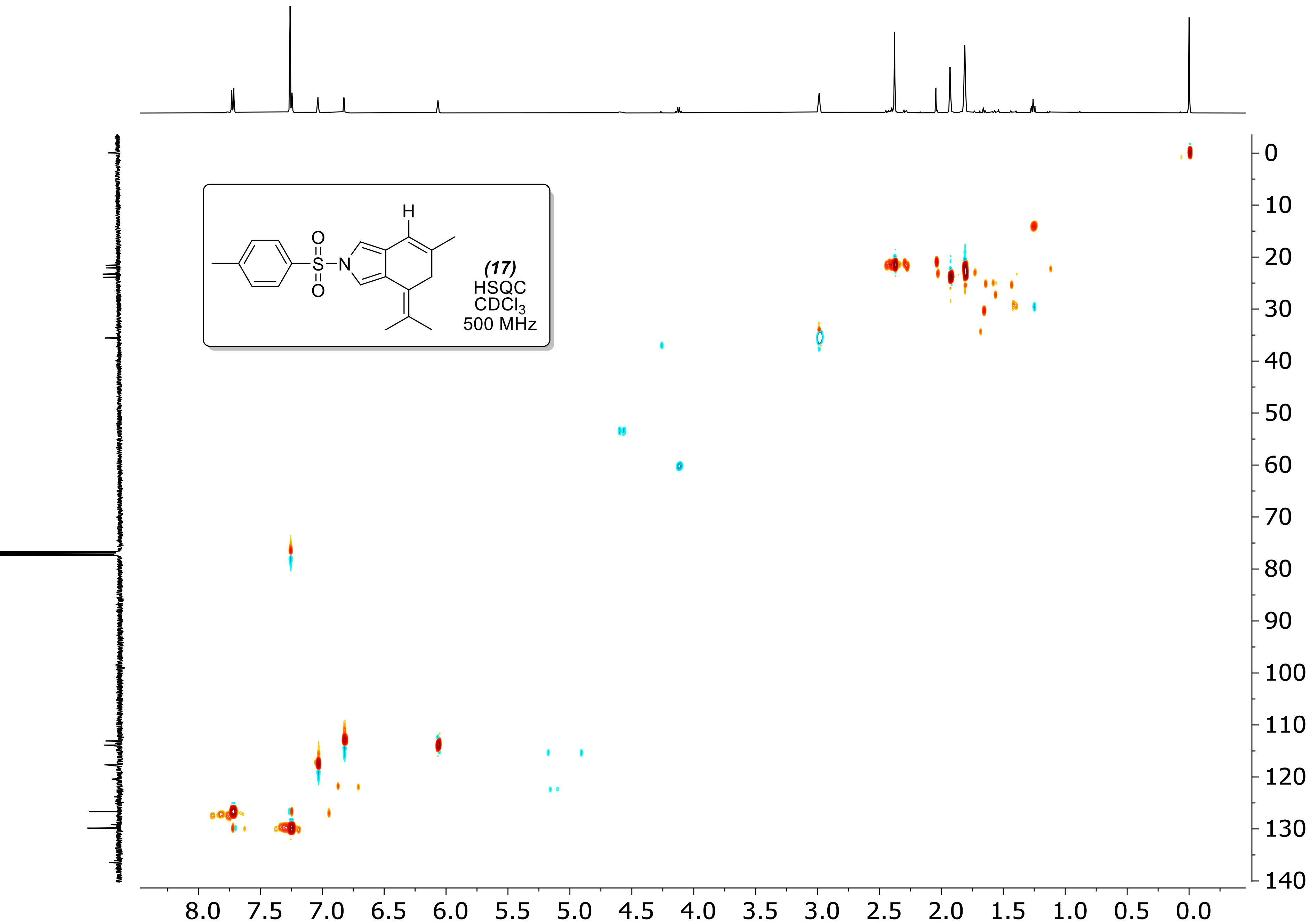
H
O
S
N
O
(17)
HSQC
CDCl3
500 MHz
0
10
20
30
40
50
60
70
80
90
100
110
120
130
140
8.0
7.5
7.0
6.5
6.0
5.5
5.0
4.5
4.0
3.5
3.0
2.5
2.0
1.5
1.0
0.5
0.0

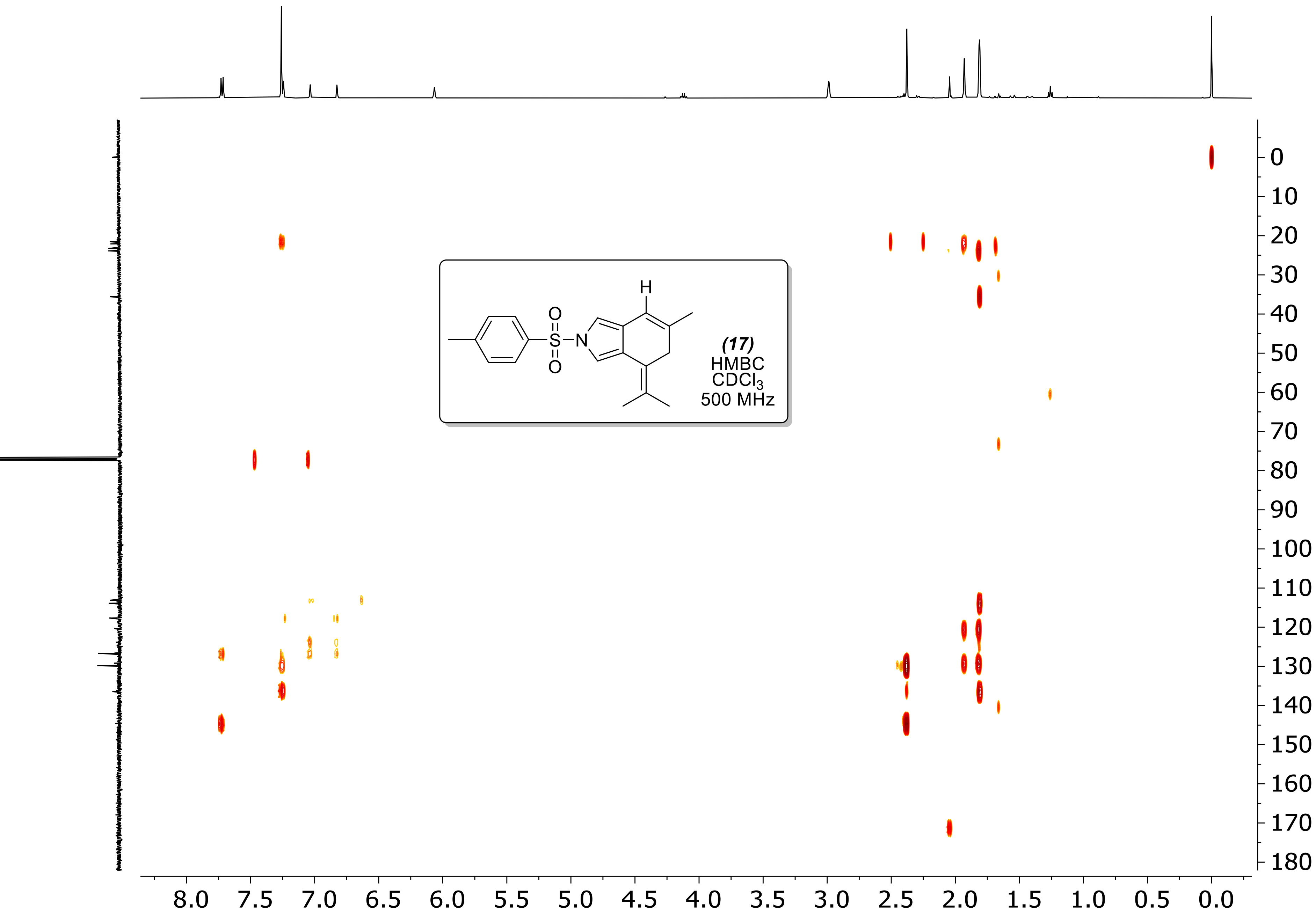
H
O
S
N
O
(17)
HMBC
$CDCl_3$
500 MHz
8.0 7.5 7.0 6.5 6.0 5.5 5.0 4.5 4.0 3.5 3.0 2.5 2.0 1.5 1.0 0.5 0.0
0 10 20 30 40 50 60 70 80 90 100 110 120 130 140 150 160 170 180

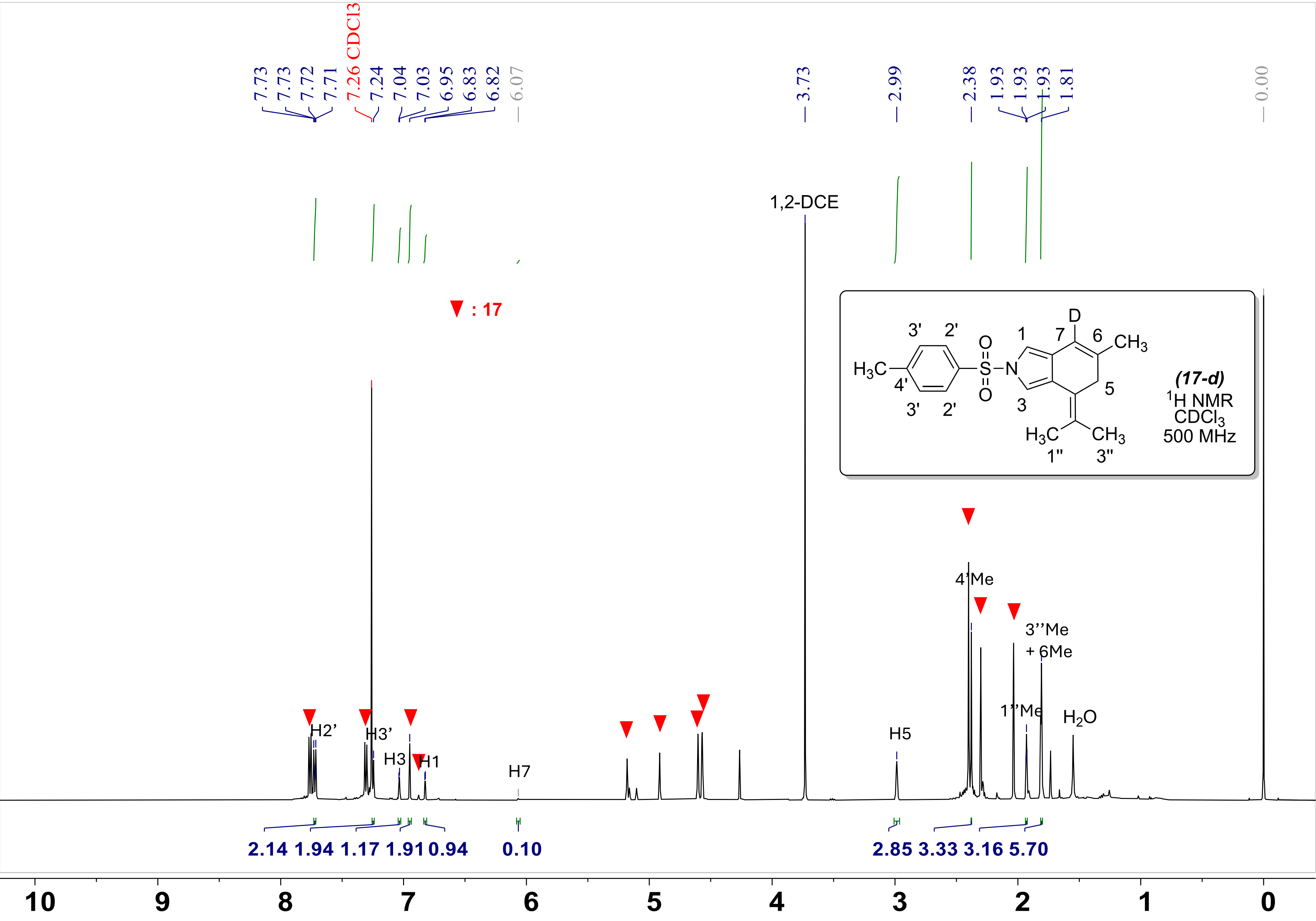
7.73
7.73
7.72
7.71
7.26 CDCl3
7.24
7.04
7.03
6.95
6.83
6.82
6.07
3.73
2.99
2.38
1.93
1.93
1.93
1.81
0.00
1,2-DCE
▼ : 17
H2'
H3'
H3
H1
H7
H5
4'Me
3''Me
+ 6Me
1''Me
H2O
(17-d)
1H NMR
CDCl3
500 MHz
2.14
1.94
1.17
1.91
0.94
0.10
2.85
3.33
3.16
5.70
10
9
8
7
6
5
4
3
2
1
0

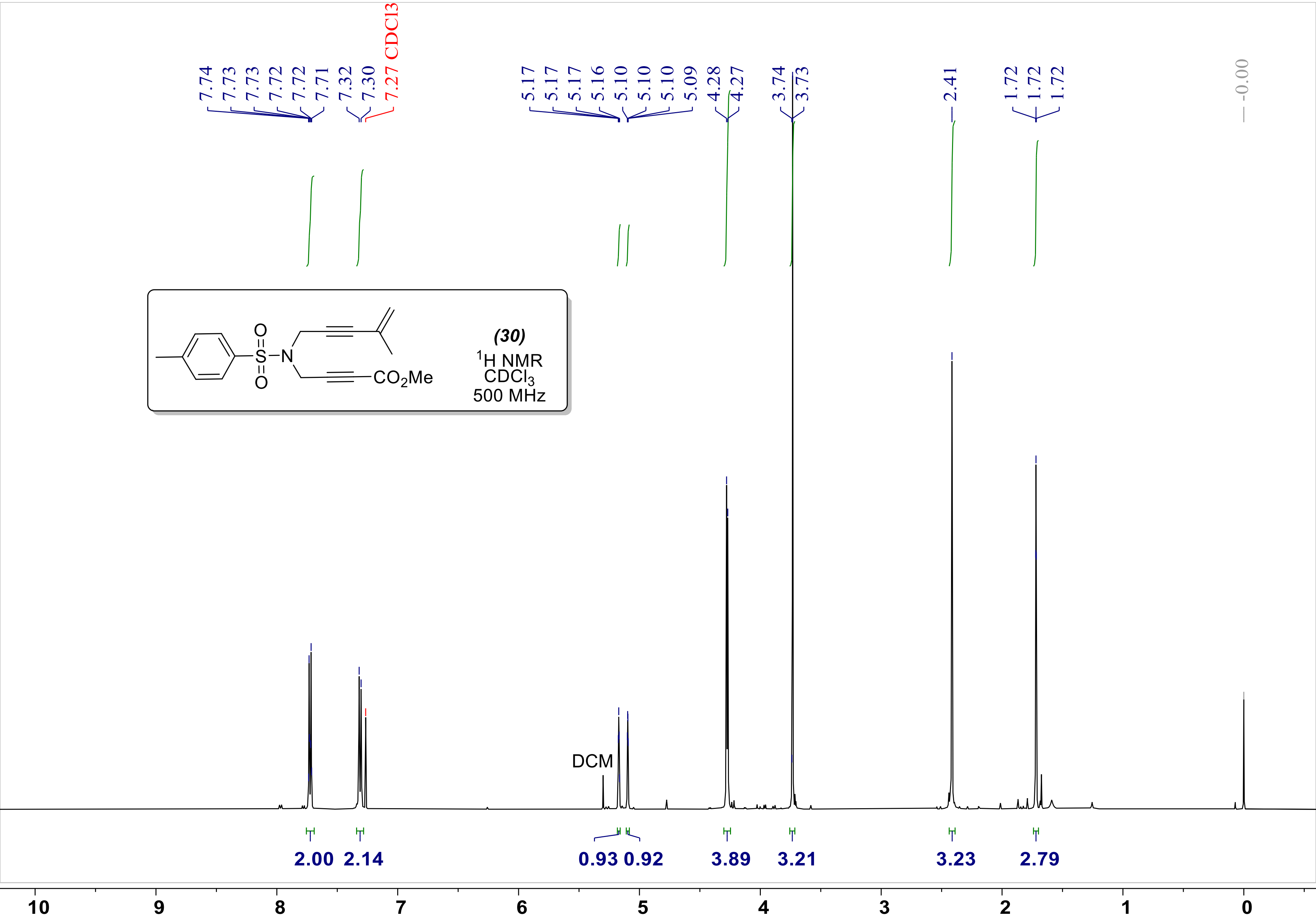
7.74
7.73
7.73
7.72
7.72
7.71
7.32
7.30
7.27 CDCl3
5.17
5.17
5.17
5.16
5.10
5.10
5.10
5.09
4.28
4.27
3.74
3.73
2.41
1.72
1.72
1.72
-0.00
O
S
N
O
CO2Me
(30)
1H NMR
CDCl3
500 MHz
DCM
2.00
2.14
0.93
0.92
3.89
3.21
3.23
2.79
10
9
8
7
6
5
4
3
2
1
0

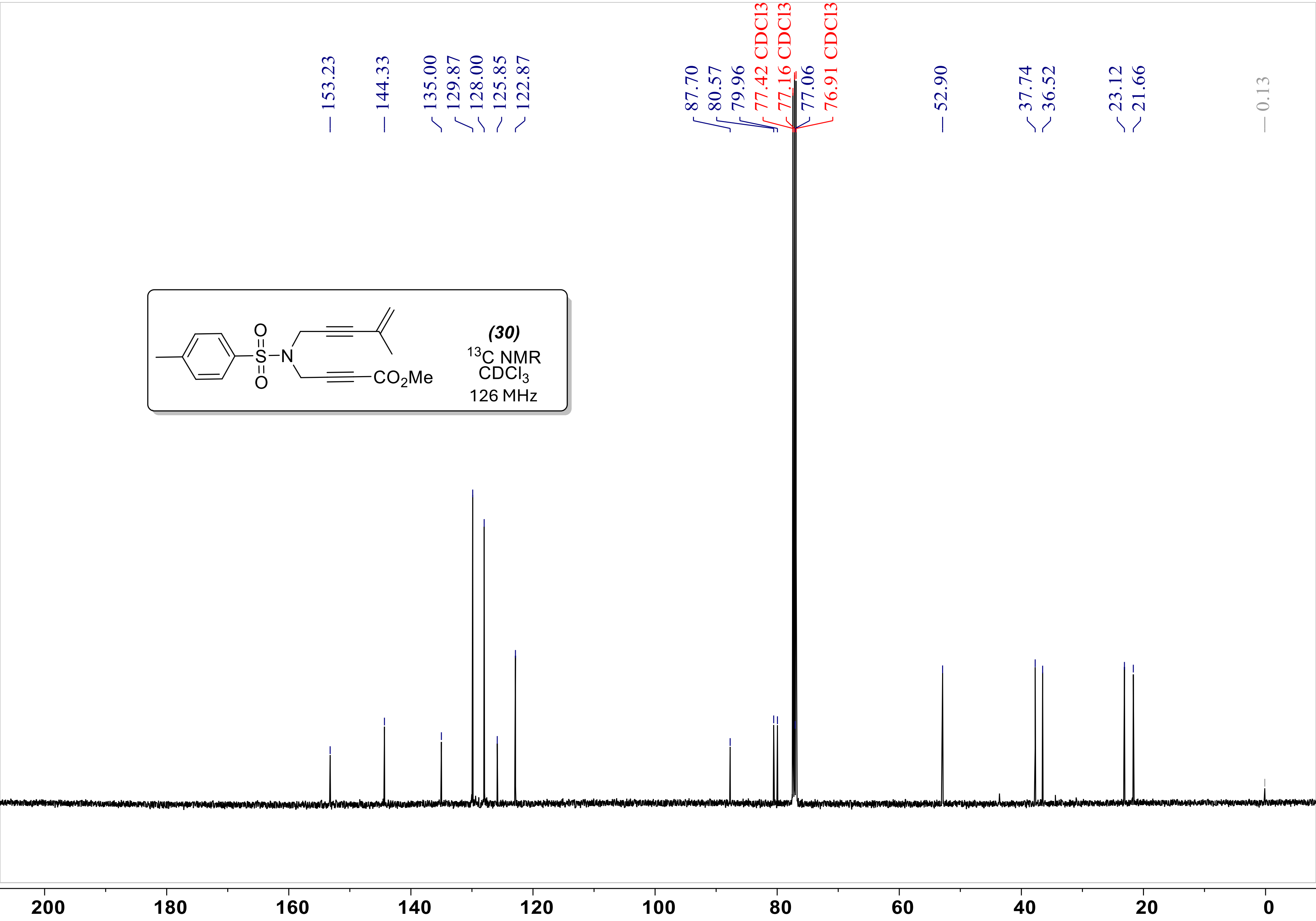
153.23
144.33
135.00
129.87
128.00
125.85
122.87
87.70
80.57
79.96
77.42 CDCl3
77.16 CDCl3
77.06
76.91 CDCl3
52.90
37.74
36.52
23.12
21.66
0.13
O
S
N
O
CO_2Me
(30)
^{13}C NMR
$CDCl_3$
126 MHz
200
180
160
140
120
100
80
60
40
20
0

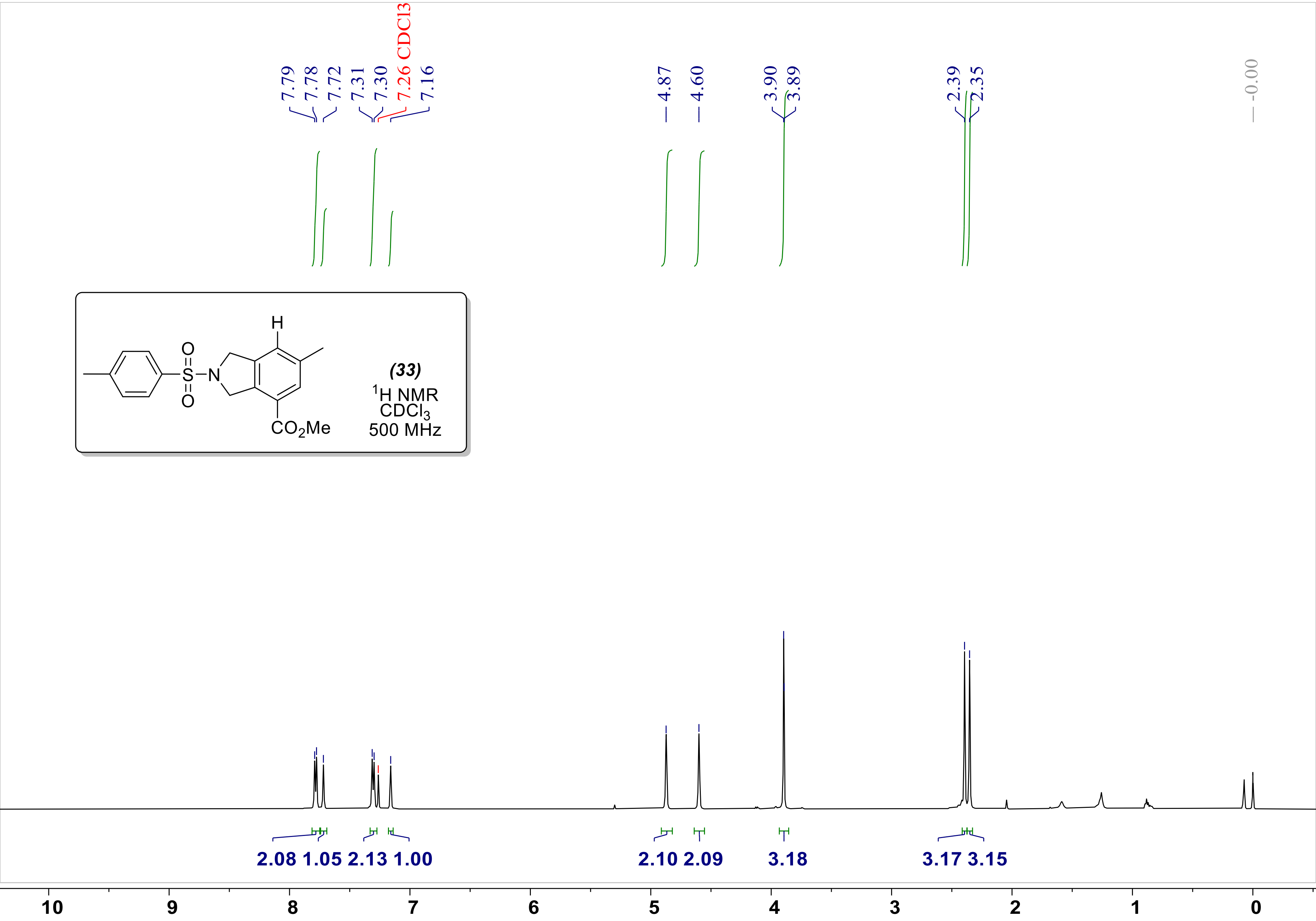
7.79
7.78
7.72
7.31
7.30
7.26 CDCl3
7.16
4.87
4.60
3.90
3.89
2.39
2.35
-0.00
H
O
S
N
O
CO2Me
(33)
1H NMR
CDCl3
500 MHz
2.08 1.05 2.13 1.00
2.10 2.09
3.18
3.17 3.15
10
9
8
7
6
5
4
3
2
1
0

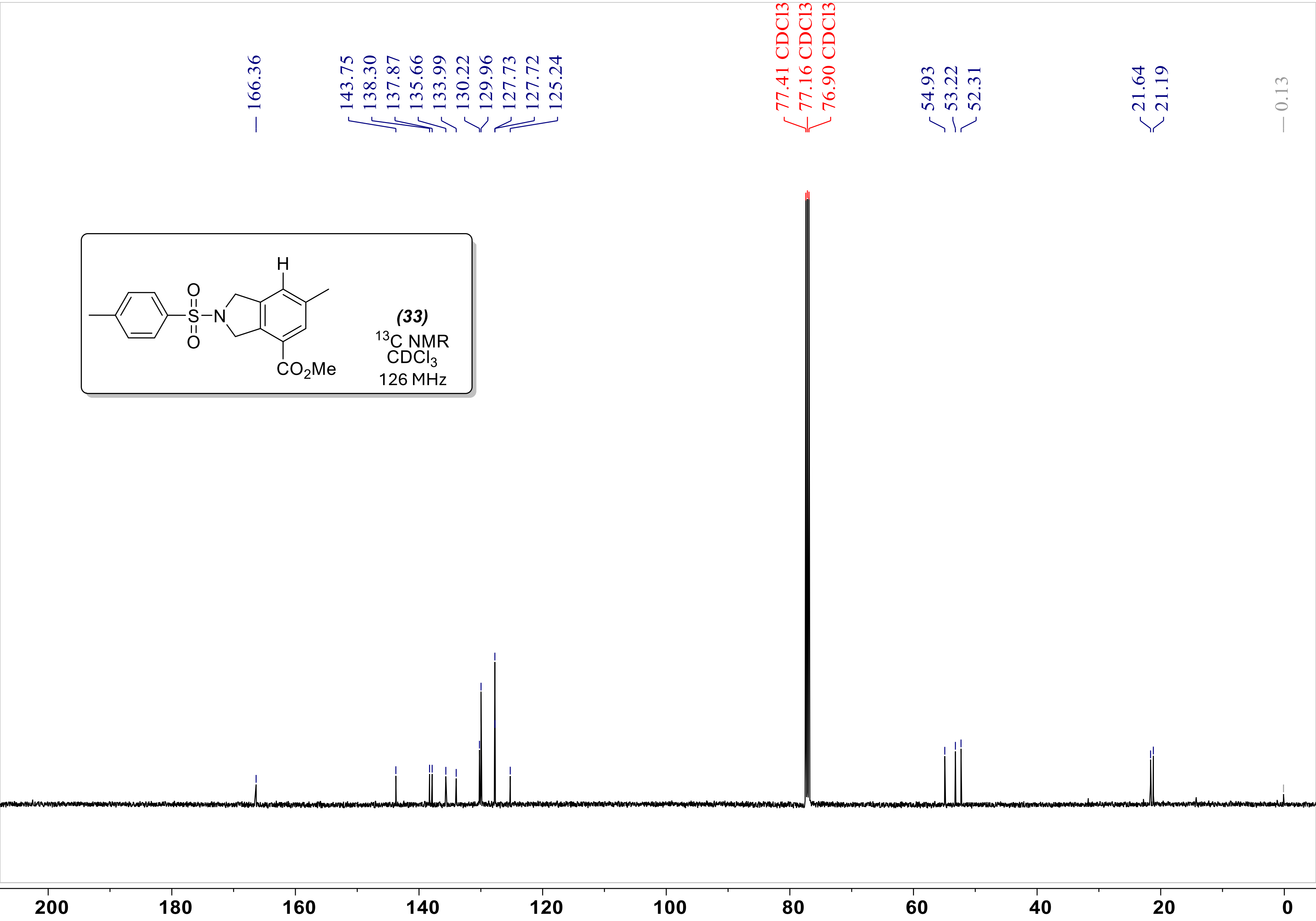
166.36
143.75
138.30
137.87
135.66
133.99
130.22
129.96
127.73
127.72
125.24
77.41 CDCl3
77.16 CDCl3
76.90 CDCl3
54.93
53.22
52.31
21.64
21.19
0.13
H
O
S
N
O
CO2Me
(33)
13C NMR
CDCl3
126 MHz
200
180
160
140
120
100
80
60
40
20
0

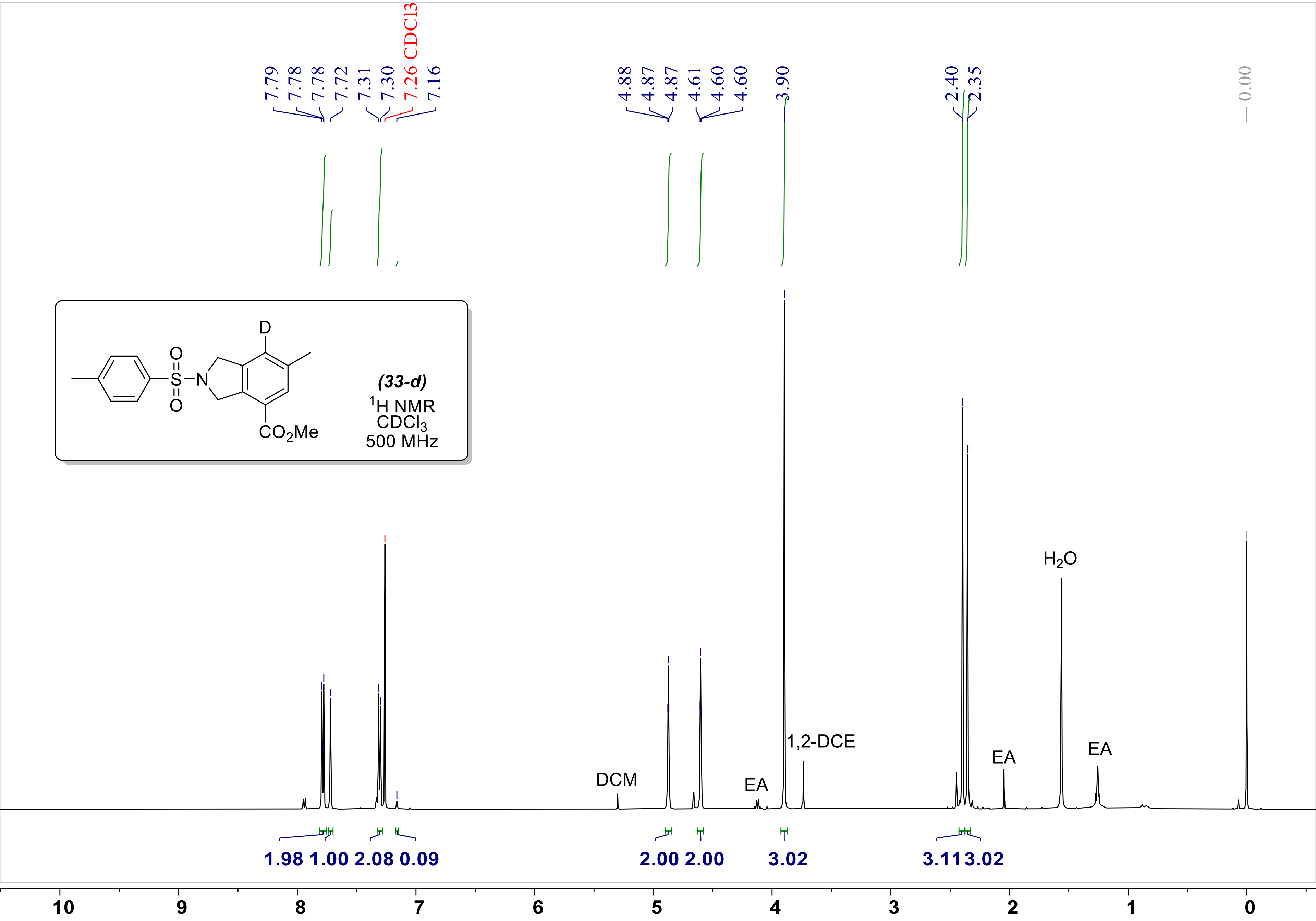
7.79
7.78
7.78
7.72
7.31
7.30
7.26 CDCl3
7.16
4.88
4.87
4.87
4.61
4.60
4.60
3.90
2.40
2.35
0.00
D
O
S
N
O
CO_2Me
(33-d)
^1H NMR
$CDCl_3$
500 MHz
H_2O
DCM
EA
1,2-DCE
EA
EA
1.98 1.00 2.08 0.09
2.00 2.00
3.02
3.11 3.02
10
9
8
7
6
5
4
3
2
1
0

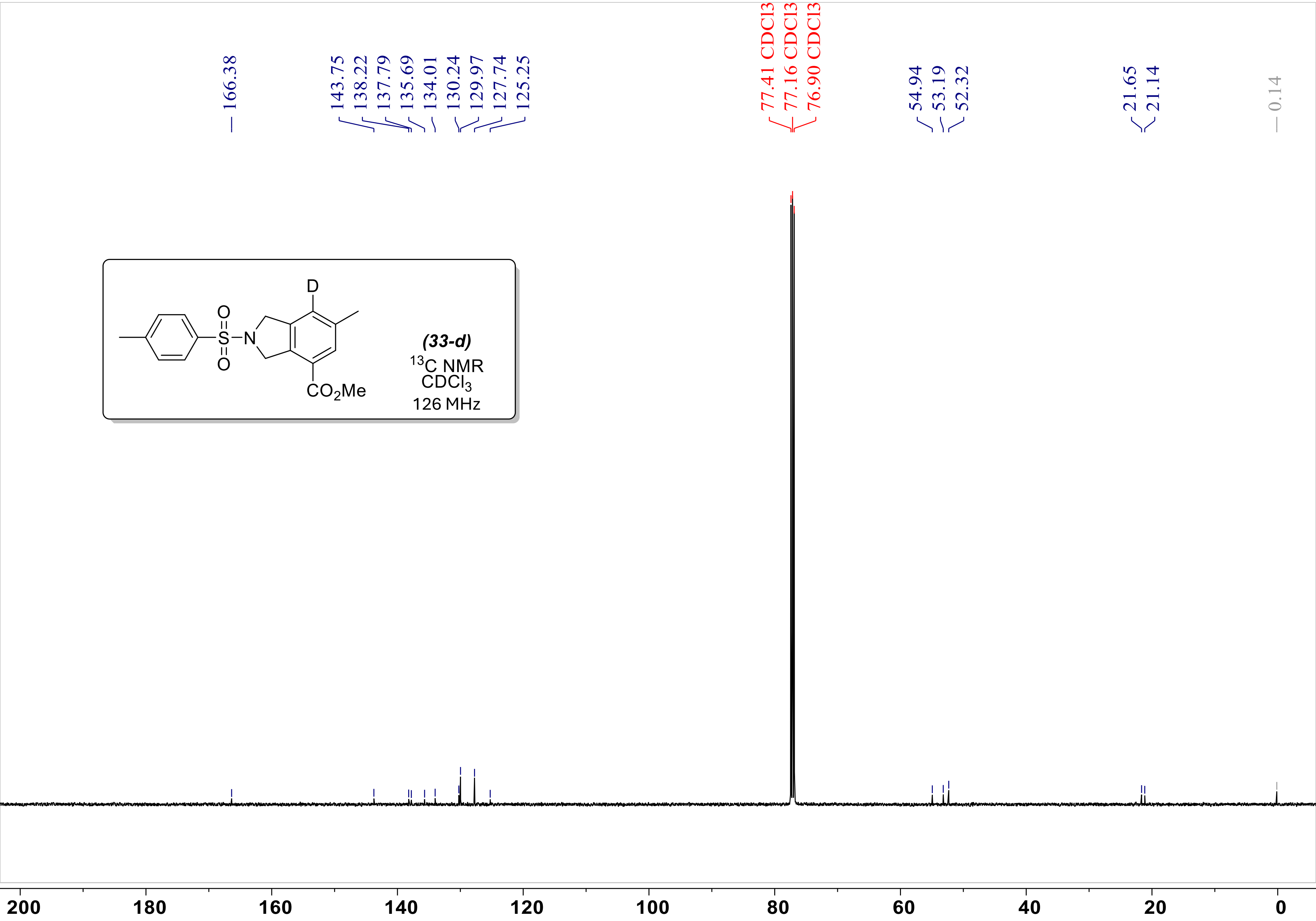
166.38
143.75
138.22
137.79
135.69
134.01
130.24
129.97
127.74
125.25
77.41 CDCl3
77.16 CDCl3
76.90 CDCl3
54.94
53.19
52.32
21.65
21.14
0.14
D
O
S
N
O
CO2Me
(33-d)
13C NMR
CDCl3
126 MHz
200
180
160
140
120
100
80
60
40
20
0
